# Mind the Energy Performance Gap: testing the accuracy of building Energy Performance Certificates in Ireland

**DOI:** 10.1007/s12053-021-09960-1

**Published:** 2021-07-17

**Authors:** Bryan Coyne, Eleanor Denny

**Affiliations:** Department of Economics, Arts Building, Trinity College Dublin, Dublin, Ireland

**Keywords:** Residential energy use, Consumer information, Modelling error, Energy performance certificate, Q40, Q48, D83

## Abstract

Ireland’s Climate Action Plan aims upgrade 500,000 homes to B2 Energy Performance Certificate (EPC) standard by 2030. Evidence of an Energy Performance Gap, where actual energy use differs from the EPC, could undermine progress towards such targets. This paper studies the energy performance gap for a general housing sample (*n* = 9923) over multiple years. It provides a novel comparison between whole-home energy use (electricity and gas) that accounts for fuel switching and removes potential rebound effects by excluding households that may have changed their behaviour following a retrofit. Results suggest that actual energy use is unresponsive to the EPC, with a range of 457 kWh/year observed across EPC-level averages for the entire sample. This difference equated to less than 5% of the sample average annual energy use observed. The Energy Performance Gap range features an average deficit of 17% below theoretical energy use. The least energy efficient dwellings feature an average difference ranging from − 15 to − 56% of the relevant EPC. Conversely, energy efficient houses display higher-than-theoretical energy use, with average surpluses ranging from 39 to 54% of the relevant EPC. Results sound a note of caution for policymakers that rely on a theoretical EPC to deliver real energy savings. Future EPCs could be improved by incorporating historical household energy usage to help improve models.

## Introduction

### Residential energy policies

Approximately 75% of buildings do not meet energy efficient standards as defined by the EU building standards (European Commission 2019a). This is likely because 35% of the European dwelling stock is over 50 years old (BPIE 2011) and only 0.4–1.2% of the building stock is renovated annually, depending on the member country (European Commission 2019a). The residential sector represented 25.4% of final energy use in the EU in 2016, with the majority of energy (79.2%) used for space and water heating (Eurostat, 2019a).

The EU has set targets for renewable generation, emissions reduction, and energy efficiency to achieve climate neutrality by 2050 (European Commission 2019b). 2030 climate targets include (i) sourcing 32% of the energy mix from renewable sources, (ii) reducing GHG emissions by 40% from 1990 levels and (iii) a 32.5% improvement in energy efficiency, relative to a 2007 forecast (European Parliament, 2018). Improving energy efficiency is viewed as a key way to reduce emissions, representing almost 40% of the potential for reducing greenhouse gases for less than €60 per metric tonne of carbon dioxide equivalent (McKinsey, 2010).

The EU Energy Performance of Buildings Directive (EPBD) is a regulation that aims to improve building energy efficiency in member states (European Commission 2019a). It emphasises the use of Energy Performance Certificates (EPCs) in building sale and rental advertisements (European Commission 2018) to improve information for buyers and sellers on the indicative energy performance of a building. EPCs also contribute towards other aspects of the EPBD, such as providing guidance on possible energy efficiency improvements.^1^ In Ireland, the Climate Action Plan plans to reduce energy use in buildings through a policy to upgrade 500,000 homes to an energy efficient B2 standard (Government of Ireland, 2019). This is equivalent to a quarter of the national dwelling stock (Central Statistics Office, 2017).

### The value of Energy Performance Certificates

Despite policymaker enthusiasm for introducing EPCs, evidence on the relationship between EPCs and property prices is mixed. Although studies for the EU and Ireland found correlations between a better rating and a higher sales or rental prices in EU countries (European Commission, 2013; Hyland et al., 2013), German homeowners found it was difficult to translate EPCs into the value of energy efficiency and did not consider it a priority in their property purchase decision (Amecke, 2012). Evidence from Northern Ireland that applies quantile regression finds evidence of a premium attached to energy efficient dwellings at high sales prices and discounts attached to low energy efficiency dwellings for sale at high prices (McCord et al., 2020).

EPCs have also been related to other important outcomes. Comerford et al. (2018) find that introducing an EPC induced investment in household energy efficiency in the UK. Evidence from Wales suggests a statistically significant price premium of 12.8% for A/B-rated dwellings (Fuerst et al., 2016). However, the authors make an important observation that energy performance may not be the only factor driving this price premium, as it is likely to be correlated with other desirable factors.

### Limitations of Energy Performance Certificates

The mixed evidence on the value of EPCs is unsurprising. Evidence for the Irish EPC suggests that trust in the measure could be undermined due to systematic bunching^2^ in the distribution of EPCs with regard to property sale prices (Hyland et al., 2016). Furthermore, EPCs have been shown to suffer from a lack of ex-post verification between measured and theoretical energy use (Burman et al., 2014; van Dronkelaar et al., 2016).

There is often a disparity between the engineering model-based EPC and actual energy use (Cozza et al., 2020; De Wilde, 2014; Gram-Hanssen & Georg, 2018; Majcen et al., 2013; Zou et al., 2018). This is known commonly as the Energy Performance Gap (EPG).^3^ Research has found a negative relationship between dwelling energy efficiency and the direction of the EPG, with a positive EPG (higher energy use) for energy efficient dwellings and a negative EPG (lower energy use) for the least efficient (Cozza et al., 2020; Majcen et al., 2013; van den Brom et al., 2018). Studies of the EPG have identified the influential role of occupant behaviour (De Wilde, 2014; Gram-Hanssen & Georg, 2018; Zou et al., 2018).

There has been substantial evidence on the behavioural factors influencing energy use when dwelling energy efficiency changes (‘retrofit’). Many studies have identified ‘rebound’ effects, where a lower effective price of heating encourages increased energy use (Heesen & Madlener, 2018; Sorrell et al., 2009). Some studies of retrofit have found a ‘prebound’ effect, where the least energy efficient dwellings consume less heating energy than expected (per their EPC) following a retrofit (Sunikka-blank & Galvin, 2012). Accurate estimates of the EPG are complicated by improvements in dwelling energy efficiency that may induce any behavioural change in the occupant. Aydin et al. (2017) show a negative relationship between household income and rebound in gas use.^4^ Research into an Irish energy retrofit also found that socially vulnerable occupants often under-heat their homes and use more energy and alternative heating fuels following a retrofit (Coyne et al., 2018).

The rapid nature of technological change also poses challenges for research into the EPG. Delghust et al. (2015) note how research needs to study all fuels used in the house, including electricity, which represents a greater share of energy use in efficient dwellings and is becoming more popular due to changes in heating systems.

### Contribution

Evidence about the EPG suggests that policies aiming to reach a certain EPC standard may not deliver the expected energy savings (Cozza et al., 2020; Gram-Hanssen & Georg, 2018; Zou et al., 2018). Research has noted that country-level differences in the implementation of the EPBD require country-specific studies of the Energy Performance Gap (Andaloro et al., 2010; Delghust et al., 2015). This is especially true for Ireland, where there are ambitious plans to upgrade the energy efficiency of the dwelling stock (Government of Ireland, 2019).

This is the first paper that tests for the presence of an EPG using a measure of whole-home energy use for a non-social housing sample of 9923 households that do not receive a retrofit. The key contribution of this paper is the combination of (i) the analysis of whole-home energy use, (ii) for a non-social housing sample that (iii) does not feature behavioural changes that would be induced by retrofit. Previous studies have considered one or two of these aspects, but this is the first study to combine all three and overcomes some of the limitations of previous studies. The following three paragraphs detail the three main contributions of the study:

i) This estimate of the EPG considers a whole-home measure of energy use. This is different to other studies which focus exclusively on the EPG for a single fuel source for space and water heating (Cozza et al., 2020; van den Brom et al., 2018). Monitoring both electricity and gas demand can capture fuel switching behaviour. This is important due to the increasing use of alternative fuels in households, especially for socially vulnerable homes in low efficiency dwellings (Coyne et al., 2018; van den Brom et al., 2018). Accounting for electricity use is especially relevant as energy efficient dwellings tend to have a higher share of heating from electricity (Delghust et al., 2015). Failure to account for fuel switching may overstate the true EPG when measured using only one fuel source.
ii) This research features a generally representative sample of households over a 2-year period. Some earlier EPG studies only feature social housing tenants (Majcen et al., 2013; van den Brom et al., 2018), a cohort which has been shown elsewhere to have particular energy use behaviour (Coyne et al., 2018; Delghust et al., 2015). For this reason, a general sample of households may provide a more general view of the EPG.
iii) The estimate of the EPG does not include changes in occupant behaviour that would be induced due to a change in dwelling energy efficiency from a retrofit. Other studies note the potential for retrofit in their sample used to estimate the EPG (Cozza et al., 2020; Heesen & Madlener, 2018). However, research has shown that a retrofit can induce a behavioural change in energy use (Sorrell et al., 2009; Sunikka-blank & Galvin, 2012; Webber et al., 2015). This is the first study to estimate the EPG for a sample that do not receive a grant supported retrofit during the observation period, while addressing a need for residential modelling with usage data (SEAI 2018).^5^

The paper is laid out as follows: Section Literature details select relevant literature and background on the Irish case study. Section Methodology and Data details the methodology, data and variables used. Section Results presents results, while Section Discussion discusses the main findings. Section Conclusion concludes with some policy recommendations.

## Literature

### Studies of the Energy Performance Gap

The Energy Performance Gap (EPG) is central to this study. As noted in Section Limitations of Energy Performance Certificates, there is a diverse range of studies of the difference between actual energy use and the level calculated by an EPC. Differences between the engineering model-based EPC and actual energy use often arise (Cozza et al., 2020; De Wilde, 2014; Gram-Hanssen & Georg, 2018; Majcen et al., 2013). The different implementations of the EPBD across member states justify the need for country-specific research (Andaloro et al., 2010; Delghust et al., 2015).

The EPG often has a distributional aspect, where households in buildings of calculated poor energy efficiency and socially vulnerable occupants demonstrate substantial under-consumption, relative to the EPC (Cozza et al., 2020). Studies of the EPG for a sample of social housing tenants found that energy efficient dwellings use more energy than calculated and vice versa (Majcen et al., 2013; van den Brom et al., 2018).

Studies of the EPG are complicated by behavioural changes in the occupant (‘rebound’) observed due to retrofit, where a lower effective price of heating encourages increased energy use (Heesen & Madlener, 2018; Sorrell et al., 2009). In some cases, a retrofit has been shown to lead to a fall in energy use (Sunikka-blank & Galvin, 2012). Aydin et al. (2017) highlight a negative relationship between household income and rebound in gas use, with the lowest income quintile featuring an average rebound almost ten percentage points higher than the average rebound for the rest of the distribution.

Estimates of the EPG are further complicated if improvements in building energy efficiency from a retrofit do not deliver the expected improvement (Gram-Hanssen & Georg, 2018). In the UK, Dowson et al. (2012) note that model predicted energy savings may be halved in reality due to poor installation, monitoring and increased heating use post-retrofit. Research into an Irish energy retrofit also found that socially vulnerable occupants often under-heat their homes and use alternative heating fuels (Coyne et al., 2018).

Many studies of the EPG find occupant behaviour to be an important factor (De Wilde, 2014; Gram-Hanssen & Georg, 2018; Zou et al., 2018). In a commercial context, actual energy use can be 2.5 times larger than predicted (Menezes et al., 2012). Herrando et al. (2016) find an average EPG of 30%. Majcen et al. (2013) find that energy inefficient homes consume less than predicted and energy efficient homes consume more than predicted for a sample of 200,000 social housing tenants in the Netherlands. Van den Brom et al. (2018) find similar results for a larger sample of Dutch social housing tenants. They also find that dwelling type is more responsible for the discrepancy in actual energy use than the EPC.

Most studies only consider energy used for space and water heating and do not account for potential fuel switching, which has been shown for select cohorts (Coyne et al., 2018; Delghust et al., 2015). Although research has identified discrepancies between the actual and theoretical level of energy use, this message has not reached policymakers (Gram-Hanssen & Georg, 2018). Reasons for this discrepancy include the limitations of building modelling, inaccurate assumptions regarding occupant behaviour, and flaws during the building design phase.

In summary, research has shown that modelling residential energy use is challenging. Part of this challenge arises from how occupant behaviour changes over time through rebound effects from changes in building energy efficiency. The EPG has also been shown to be particularly sensitive to the socioeconomic status of occupants. For these reasons, a study of the EPG using a measure of whole-home energy use for a general sample of households that did not receive a retrofit is highly relevant.

### Ireland as a case study

Ireland intended to improve energy efficiency (lowering energy use) by 20% before 2020, relative to average national energy use from the period 2001 to 2005. This equates to energy savings of 31,925 GWh (DCENR 2009). As part of the EU Energy Efficiency Directive, member states must submit a National Energy Efficiency Action Plan with specific measures designed to improve energy efficiency (European Union, 2012). By early 2017 Ireland had only achieved a 12% improvement in energy efficiency and is expected to miss the 2020 target by 3.77% (DCCAE, 2017). Achieving compliance for the 2020 target could cost €80–140 million.^6^

Despite this, Ireland has made progress in improving residential energy efficiency. Energy use per dwelling has fallen by 32% from 1990 to 2015 due to technology improvement, retrofits, building regulations and macroeconomic factors (SEAI 2016). This reduction is 37% when correcting for climate during the period. However, there is more to be done as Ireland has the fourth highest level of greenhouse gas emissions in the EU of 13.3 tonnes of CO_2_ equivalent per capita in 2017 (Eurostat, 2020).

Irish homes consume the most energy on average in the EU, with the second largest average occupancy in the EU-28 of 2.7 persons per house (SEAI 2018). According to EU-SILC data from 2017, 8.3 percent of the Irish population live in apartments (Eurostat, 2019b), lower than the EU average of 41.9 percent and almost half the second-lowest ranked country, the UK (14.7 percent). Electricity plays an important role in residential energy use. In 2017, over 20 percent of electricity used in the Irish residential sector was for space and water heating.^7^

Irish interventions to improve residential energy efficiency aim to simplify consumer decision-making for durable appliances (Carroll et al., 2016), to improve dwelling energy efficiency through a grant-supported retrofit (Scheer et al., 2013) or to alter intraday electricity (Di Cosmo et al., 2014) and gas (Harold et al., 2018) usage patterns. Research has established how information from an EPC on theoretical dwelling energy efficiency is positively associated with property sale and rental prices (Hyland et al., 2013). Lastly, Hyland et al. (2016) suggest there is room to improve the Irish EPC due to systematic bunching in the distribution of ratings.

Evidence of an EPG presents an issue if policymakers expect real emissions reductions from improving the dwelling stock to a certain EPC threshold. In Ireland, the government aims to retrofit 500,000 homes to B2 EPC standard by 2030 (Government of Ireland, 2019). The presence of an EPG would cause actual savings to deviate from the level expected.

## Methodology and data

### Methodology

Policymakers attempting to reduce emissions by upgrading the dwelling stock to a certain EPC standard face a problem if an EPC is based on assumptions regarding theoretical occupant energy use (${TQ}_{i}$) that does not accurately reflect actual occupant energy use $({AQ}_{it})$. Consequently, the presence of an Energy Performance Gap (EPG) may limit the effectiveness of policies designed to lower residential energy use by targeting a benchmark EPC standard. This paper features three distinct research questions that explore the existence of an EPG and the factors influencing actual energy use. Each research question directly corresponds to a subsection of the results.

The first research question (Section Annual results) tests for the presence of an Energy Performance Gap with a null hypothesis that the EPC accurately reflects actual occupant usage (Eq. 1). For a given household *i* in year *t*, actual household energy use (AQ_it_) is equal to the theoretical EPC level of energy use (TQ_i_) if there is no Energy Performance Gap. Since the EPC is an estimate that does not account for appliance use and occupant behaviour, it will not reflect true dwelling energy use (discussed in Section Dependent Variable: Actual Energy Use). It is expected that this difference will not be equal to zero (Cozza et al., 2020; van den Brom et al., 2018; Zou et al., 2018). This result is presented in aggregate (kWh/year) and as a percentage of the EPC (Cozza et al., 2020). Equation 1 shows testing for the presence of an Energy Performance Gap.

1 $$\mathbf{H}0: {\mathbf{A}\mathbf{Q}}_{\mathbf{i}\mathbf{t}} - {\mathbf{T}\mathbf{Q}}_{\mathbf{i} }=0$$

This question is highly relevant since the Energy Performance Gap is widely accepted in the research community but often ignored in policy discourse (Gram-Hanssen & Georg, 2018). This is the first study that controls for whole home energy use, the sample and the potential for retrofit-induced behavioural changes.

The second research question (Section Bimonthly results) aims to quantify the extent to which key dwelling factors influence actual energy use at the bimonthly level (*n* = 149,518 readings). It uses a linear regression at the bimonthly time frequency and accounts for the influential role of seasonality in energy use. It considers the EPC and relevant dwelling characteristics (detailed in Sections. The Irish EPC and Dwelling Characteristics and Weather, respectively). It models actual energy use (AQ_it_) for household *i* in period *t* as a function of the theoretical energy efficiency of the dwelling (TQ_i_), a vector (X_i_) of key dwelling features such as dwelling type, size, number of stories, age and a vector (W_t_) of time-varying weather controls (Eq. 2). Results (Section Bimonthly results) begin by regressing actual energy use on the fully interacted EPC (Model 1) and then expand to include dwelling characteristics (Model 2) and a time fixed effect (Model 3). Equation 2 shows linear regression of actual energy use at bimonthly frequency.

2 $${\mathrm{AQ}}_{\mathrm{it}}={\mathrm{a}}_{\mathrm{i}}+{\upbeta }_{1}{\mathrm{TQ}}_{\mathrm{i}}+{\upbeta }_{2}{\mathrm{X}}_{\mathrm{i}} + {\upbeta }_{3}{\mathrm{W}}_{\mathrm{t}}+{\mathrm{u}}_{\mathrm{it}}$$

The third research question (Section Bimonthly results: Split by EPC) explores whether the relationship between actual energy use and key dwelling factors persists across each of the five EPC bands *j* (Eq. 3). This is motivated by the potentially different influence of covariates across the EPC spectrum. This relationship is explored for each specific EPC grade at the bimonthly frequency (Model 4) using the linear regression used in Model 3, featuring a time fixed effect. Equation 3 shows linear regression of actual energy use at bimonthly frequency — by EPC grade.

3 $${\mathrm{AQ}}_{\mathrm{ijt}}={\mathrm{a}}_{\mathrm{i}}+{\upbeta }_{1}{\mathrm{TQ}}_{\mathrm{ij}}+{\upbeta }_{2}{\mathrm{X}}_{\mathrm{i}} + {\upbeta }_{3}{\mathrm{W}}_{\mathrm{t}}+{\mathrm{u}}_{\mathrm{it}}$$

### Data sources

Household energy use data of electricity and natural gas (A) is sourced from Electric Ireland, the largest residential electricity utility in Ireland. This paper studies homes with natural gas heating observable by meter readings. This data is observed from November 2014 to June 2017, sixteen bimonthly^8^ periods. This is merged with dwelling information from SEAI (B) using the common meter point number. Time-varying weather (C) controls are also included (Table 1). The sample consists of 9923 homes, 19,251 customer-year observations and 149,518 bimonthly readings. Appendix 2 details the data cleaning process, including the removal of 333 households with highly abnormal energy use. This did not affect later results. As in Cozza et al. (2020), such households likely represent a holiday home that is sparingly used. The sample distinguishes between households that never receive a retrofit (*n* = 8311) and households that receive one prior to the observation period (*n* = 1612 houses).^9^

**Table 1:** Data sources

ID	Data	Details	Source
A	Energy use	Electricity and gas readings	Electric Ireland
B	Building Energy Rating	Dwelling features, EPC	SEAI
C	Weather	Heating degree days (HDDs), rainfall	Met Eireann

Appendix 2 details the data cleaning process and the handling of outliers and unreliable data

### The Irish EPC

SEAI operates the Building Energy Rating (BER) scheme, which is the Irish EPC. A BER is required for every property sold, rented or in receipt of a grant-supported retrofit (European Union, 2002). The BER denotes the theoretical energy performance of a dwelling using a 15-point scale from A1 to G in units of kilowatt-hour of primary energy per metre squared per annum (Table 2). It is compliant with the EU Energy Performance of Buildings Directive and is based on both IS EN 13,790 and the UK Standard Assessment Procedure for dwelling energy ratings (SEAI 2012). Equation 4 shows EPC primary energy components.

**Table 2:** Building energy rating (BER) levels and simplified EPC

BER	A1	A2	A3	B1	B2	B3	C1	C2	C3	D1	D2	E1	E2	F	G
	< 25	> 25	> 50	> 75	> 100	> 125	> 150	> 175	> 200	> 225	> 260	> 300	> 340	> 380	> 450
Simple EPC	AB						C			D		E		FG	
	0–150						151–224			225–299		300–379		> 380	

Source: SEAI. Note: Values in kWh/m^2^/year of primary energy. Simplified EPC is used in later analysis

4 $${TQ}_{Total}={Q}_{SpaceHeat} + {Q}_{WaterHeat} + {Q}_{AuxEnergy} +{Q}_{Lighting} -{Q}_{PV}- {Q}_{CoGen}$$

The BER calculates ‘the energy required for space heating, ventilation, water heating and lighting, less savings from energy generation technologies’ (SEAI 2012). Equation 4 details its components, which are similar to other dwelling asset rating models (Majcen et al., 2013; van den Brom et al., 2018). The BER is influenced by factors such as dwelling size, type, insulation, ventilation and heating system (SEAI 2014). It reflects theoretical primary energy use for space and water heating, ventilation and lighting.^10^ It does not include energy consumed by appliances, estimates to be roughly 20% of delivered domestic energy use (SEAI 2018).

There is no formal validation of the BER awarded from the in-home audit using real billing information. This deficiency has also been noted in studies of the UK EPC (Burman et al., 2014; van Dronkelaar et al., 2016). Collins and Curtis (2018) examine changes in BER pre- and post-retrofit and find discontinuities in the national distribution of post-retrofit BERs, but not in the pre-retrofit BERs. They find no evidence of illicit behaviour by BER assessors, but a high rate of low energy lighting (as a retrofit measure) prevalent in the distribution. This study is the first evaluation of the BER using actual energy use data for a sample without retrofit.

Weather conditions are considered at a local level, but the model assumptions regarding occupant heating behaviour are more important (SEAI 2012). The BER assumes that the heating season runs from October to May inclusive, with the primary living space being heated to 21 °C and the rest of the house heated to 18 °C for 8 h a day (SEAI 2012). Given that space and water heating demand is the single largest energy demand in the home, the a priori expectation is that differences between actual and theoretical energy use would be largely driven by deviations in actual heating behaviour, especially after accounting for appliance usage, which is not included in the EPC.

### Dependent variable: actual energy use

Most studies of the Energy Performance Gap draw a comparison between metered energy for heating with the theoretical EPC (Cozza et al., 2020; Heesen & Madlener, 2018; Majcen et al., 2013; Scheer et al., 2013). Such studies fail to capture electricity used as a secondary fuel (nor do they seek to). This omission has the potential to overstate the true Energy Performance Gap and may acutely affect the most energy efficient homes, which feature a larger share of electricity use (Delghust et al., 2015). It may also disproportionately affect homes that engage in substantial fuel switching, such as low income social housing occupants (Coyne et al., 2018). For these reasons, we include an adjustment for appliance use to allow comparability with the EPC (since the EPC does not explicitly include appliances).

This study leverages the rich data available to develop a measure of the Energy Performance Gap that compares whole-home energy use (natural gas plus electricity) with the theoretical Irish EPC, which is denoted in units of primary energy use. Table 3 summarises the primary energy factors for the sample with an average ratio of 1.24, leading to actual meter readings being inflated for comparison with the EPC. Any mention of energy from this point is referring to primary energy use, unless explicitly stated otherwise. While it would be interesting to separate primary energy consumption into electricity and gas, the EPC is an aggregate measure which does not distinguish separate consumption levels.

**Table 3:** Summary of household-level primary energy factors

	*N*	Mean	Median	SD	Min	Max	Skew	Kurt
Primary energy	9923	22,674	20,165.91	11,343.98	− 28,236.7	122,777.9	1.67	8.06
Delivered energy	9923	18,505	16,459.11	9431.09	− 27,848.8	73,646.2	1.53	6.89
Ratio (P/D)	9923	1.24	1.21	0.14	1	3.11	7.31	69.4

Source: Author’s calculations using SEAI data for 9923 households. Values in kWh/year. Delivered energy includes energy assumed to be consumed in the dwelling. Primary energy includes generation and transmission losses. The ratio helps to scale metered energy use to reflect actual energy use

In order to compare theoretical energy use from the EPC, the variable of actual energy use is adjusted to account for the heatable floor space and the ratio of primary to delivered energy (Eq. 5).^11^ Actual energy use must also be adjusted to reflect the fact that EPCs do not include energy use for appliances within the home. SEAI (2018) estimates that appliance usage comprises, on average, 20% of Irish home energy use. Results in this paper consider two versions of appliance usage (*AA*_*j*_). The first (*AA*_*Relative*_) involves a relative scaling of usage to a factor of 0.8, based on SEAI (2018). Equation 5 presents the construction of the actual energy use variable (per square metre).

5 $${AQ}_{it}=\frac{{DeliveredEnergy}_{i}}{{HeatableFloorArea}_{i}} * \frac{{PrimaryEnergy}_{i}}{{DeliveredEnergy}_{i}}*{AA}_{j}$$

**Fig. 1:**
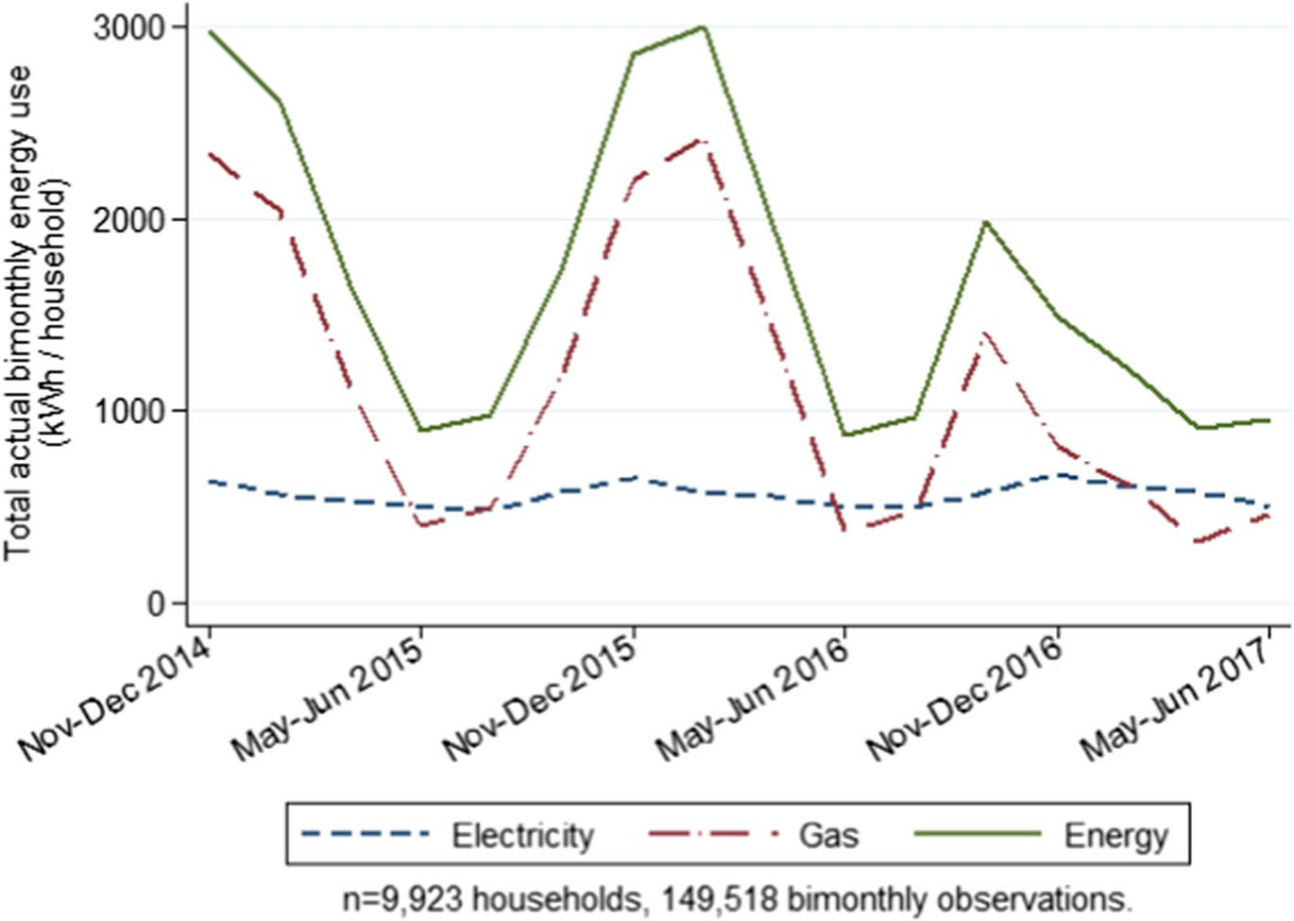
Household average bimonthly actual energy use (AARelative)

The second version of appliance adjustment accounts for concerns about the distributional effect of a relative appliance adjustment across the spectrum of building energy efficiency. The second appliance adjustment (*AA*_*Absolute*_) involves an absolute deduction for annual appliance usage (1357 kWh/year) for a subset of appliances assumed common to each home (Owen and Foreman 2012).^12^ The bimonthly panel of 9,923 households (*n* = 149,518 readings) has a completeness of 94.17%, with an average of 15.06 periods present and an average gap of 1.31 periods. Figure 1 shows the seasonal pattern of household average energy use for the entire sample.

**Fig. 2:**
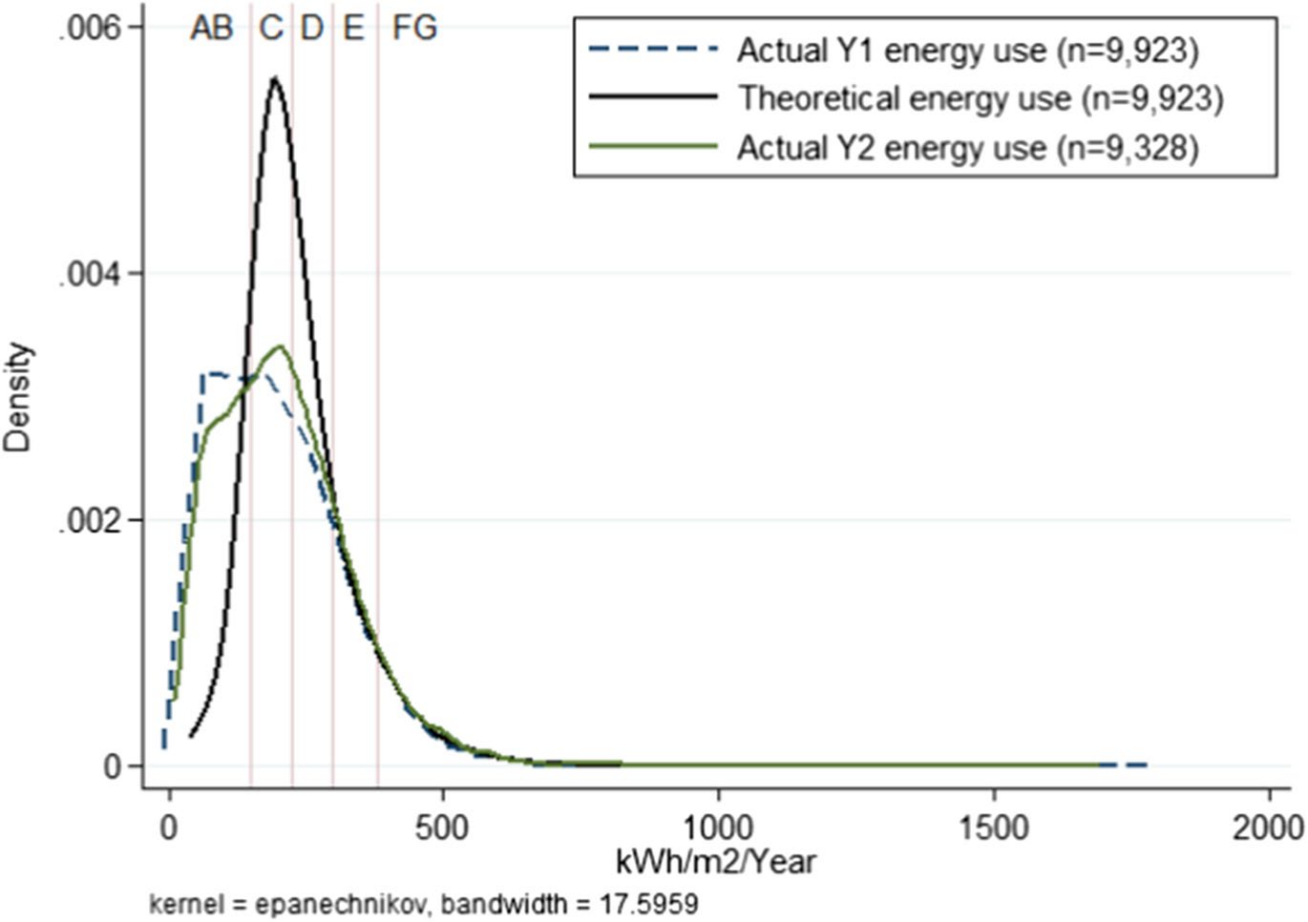
Distribution of theoretical and actual annual energy use (AARelative)

The sample features 9923 households with 1 full year of actual energy use (${\mathbf{A}\mathbf{Q}}_{\mathbf{Y}1}$) and 9328 observations with a second full year (${\mathbf{A}\mathbf{Q}}_{\mathbf{Y}2}$). Table 4 compares mean annual actual (${\mathbf{A}\mathbf{Q}}_{\mathbf{Y}1}$, ${\mathbf{A}\mathbf{Q}}_{\mathbf{Y}2}$) and theoretical (${\varvec{T}}{\varvec{Q}}$) energy use, with all variables in units of annual energy use per square metre. Figure 2 compares the distributions of annual measured energy usage (${\mathbf{A}\mathbf{Q}}_{\mathbf{Y}1}$, ${\mathbf{A}\mathbf{Q}}_{\mathbf{Y}2}$) to theoretical annual energy use (${\varvec{T}}{\varvec{Q}}$). The distribution shows a higher share of observations in the A- and B-rated (most efficient) part of the distribution and a lower share of observations in the C- and D-rated range of theoretical energy efficiency. The presence of actual energy use in the right tail of the distribution is notable, especially since the EPC lacks an upper bound on the theoretical energy efficiency of a G-rated home. A Z-test is performed for each combination of the three variables. It confirms that the three distributions are similar, with ${{\varvec{Z}}}_{{\mathbf{A}\mathbf{Q}}_{\mathbf{Y}1},{\mathbf{A}\mathbf{Q}}_{\mathbf{Y}2}\boldsymbol{ }}=0.76$, ${{\varvec{Z}}}_{{\mathbf{A}\mathbf{Q}}_{\mathbf{Y}1},{\varvec{T}}{\varvec{Q}}\boldsymbol{ }}=0.24$ and ${{\varvec{Z}}}_{{\mathbf{A}\mathbf{Q}}_{\mathbf{Y}2},{\varvec{T}}{\varvec{Q}}\boldsymbol{ }}=0.15$.

**Table 4:** Comparison of mean annual actual and theoretical energy use (AA_Relative_)

	*N*	Mean	Median	SD	Min	Max	Skew	Kurt
Actual energy use (${\mathrm{AQ}}_{\mathrm{Y}1}$)	9923	197.80	180.97	124.26	9.51	1777.77	1.50	10.80
Actual energy use (${\mathrm{AQ}}_{\mathrm{Y}2}$)	9328	211.16	197.7	123.73	11.29	1687.24	1.35	8.55
Theoretical energy use (TQ)	9923	235.44	214.54	101.03	39.97	1240.73	1.95	11.3

Values in kWh/m^2^/year

### Dwelling characteristics and weather

The SEAI dataset features dwelling information on house type, age, height and heatable floor area. Importantly, it includes a variable of theoretical energy use in units of kWh/m^2^/year, which informs the categorical EPC. Table 5 summarises key continuous variables for the sample used in later bimonthly analysis that measures the factors associated with actual energy use (Sections Bimonthly results and Bimonthly results: Split by EPC). Research has identified correlations between weather and electricity (Kavousian et al., 2013), heating (Quayle & Diaz, 1979) and appliance use (Hart & De Dear, 2004). To account for the potential influence of weather in the regression of actual energy use, households are linked at the county level with the nearest weather station.^13^ A heating degree day variable is constructed which reflects the number of days in a given period where the daily mean temperature is below 15.5 °C. This reflects days where occupants are more likely to require heating. A total bimonthly rainfall variable also features.

**Table 5:** Summary of continuous dwelling and weather control variables

Variable	Mean	SD	Min	Max
Number of floors	1.94	0.48	0	4
Year of Construction	1979	28.98	1753	2017
Percentage of home that is living area	21.33	9.81	0	81.1
Bimonthly heating degree days	53.29	10.6	10	61
Total bimonthly precipitation (in cm)	18.78	104	5.63	55.6

*n* = 9923 homes. Weather for 16 bimonthly periods and five weather stations. Living area is defined as the main living space in the household. In most cases, this is the public room with the largest floor area (SEAI 2012)

Table 6 compares key theoretical energy efficiency for the sample and the population of SEAI EPC records (which reflects roughly half of the national dwelling stock). An additional comparison by dwelling type also includes the 2016 census national occupied dwelling stock, which does not feature EPC information. SEAI data under-represents the detached dwellings and over-represents apartments and terrace homes. This is because an EPC is only required when a property is sold, leased or undergoes a retrofit. Compared to the SEAI population, the sample has a higher share of C houses and a similar share of AB and D houses. The sample under-represents detached homes and apartments and over-represents semi-detached and terraced. It is important to bear in mind the potential for the sample results to understate the true situation for FG-rated homes and to over-emphasise results for C-rated homes at a national level.

**Table 6:** Sample v SEAI population dwelling comparison

		Sample (*n* = 9923)		SEAI population (*n* = 729,599)		Occupied dwelling stock (*n* = 1,675,795)*	
		Count	%	Count	%	Count	%
EPC	AB	1351	13.61	104,084	14		
	C	4,257	42.90	270,628	37		
	D	2486	25.05	178,172	24		
	E	1059	10.67	86,401	12		
	FG	770	7.76	90,324	12		
Dwelling type	Detached	1197	12.06	232,677	32	715,133	43
	Apartment	873	8.80	144,289	20	204,145	12
	Semi-detached	3565	35.93	193,543	27	471,948	28
	Terrace	4288	43.21	159,100	22	284,569	17
	Total	9923	100	729,599	100	1,675,795	100

^*^2016 Census values from Irish CSO for occupied households, excluding ‘Not stated’ and temporary accommodation

## Results

As noted in Section Methodology, results are presented in order of the three research questions. Section Annual results quantifies the Energy Performance Gap by testing for significant differences in annual values of actual and theoretical energy use. Section Bimonthly results features regressions using the bimonthly data account for relevant covariates. Finally, Section Bimonthly results: Split by EPC studies potential heterogenous effects by EPC category. Section Dependent Variable: Actual Energy Use introduced two variants of actual energy use to account for appliance use. In each subsection, the first set of results features the dependent variable constructed using the RELATIVE appliance adjustment (AA_Relative_). The second set features the dependent variable constructed using an ABSOLUTE appliance adjustment (AA_Absolute_).

### Annual results

The sample features 19,251 annual observations of energy use (AQ), representing 9923 observations of 1 full year (${\mathrm{AQ}}_{\mathrm{Y}1}$) and a further 9328 observations featuring a second full year of energy use (${\mathrm{AQ}}_{\mathrm{Y}2}$).^14^ Energy use variables are in units of kilowatt-hours per year (kWh/year). Table 7 performs a test of paired differences showing that average annual actual energy use is significantly lower than the theoretical level from the EPC, suggesting the existence of an Energy Performance Gap (EPG). The average deficit in annual consumption is 2279 kWh, roughly 15% of the average value of 15,000 kWh/year considered by the Irish utilities regulator (CRU 2017).^15^ Differences between the sample and the regulator reference likely stem from differences in the samples. Results denote the difference between mean AQ and mean TQ (‘difference’) and the percentage difference as a percentage of the mean TQ (‘% difference’), which is similar to the measure used by Cozza et al. (2020).

**Table 7:** Difference between annual actual (AQ) and theoretical (TQ) energy use (AA_Relative_)

		Actual annual energy use (AQ)		Theoretical annual energy use (TQ)		*t*-test of equality of means			
	*n*	Mean AQ	Median AQ	Mean TQ	Median TQ	Difference		SE	*P* value
						Mean	%		
AQ_All_ – TQ	19,251	10,869	10,167	13,148	11,402	− 2279	− 17.33	61	0***
*EPC Grade*									
AB	2601	10,569	9661	7571	6620	2998	39.60	122	0***
C	8269	10,880	10,334	10,826	9734	54	0.50	70	0.44
D	4835	10,917	10,231	14,353	12,826	− 3436	− 23.94	104	0***
E	2051	11,026	10,421	18,133	16,300	− 7106	− 39.19	173	0***
FG	1495	10,964	9853	24,962	22,466	− 14,000	− 56.09	290	0***
***Dwelling***									
Apartment	1674	8115	7211	11,595	10,983	− 3481	− 30.02	163	0***
Detached	2316	13,712	13,150	19,385	17,184	− 5673	− 29.27	247	0***
Semi-detached	6905	11,398	10,917	14,008	12,495	− 2610	− 18.63	99	0***
Terrace	8356	10,197	9712	11,020	9490	− 823	− 7.47	82	0***

^***^P < 0.01, **P < 0.05, *P < 0.10. Note: Units in kWh/year. Sample features 9,923 observations of one year of actual energy use and a further 9328 observations from the same sample of houses with a second year of actual energy use. Medians reported. A test of equality of medians (using signtest STATA command (Snedecor & Cochran, 1989)) confirms the same significant differences exist as the *t*-test of means (displayed above)

The most striking observation is a distinct lack of variation in average actual energy use across the entire sample. There is only a difference of 457 kWh/year between the lowest and highest average. This suggests that the demand for energy is unresponsive to the energy efficiency of the dwelling. A similar relationship has been observed in a study of UK office buildings (Better Buildings Partnership 2019). On a comparative basis, there are significant differences between average actual and theoretical energy use. The most efficient homes (AB) feature an average positive difference of 2998 kWh per year, 39% greater than theoretical. Conversely, less efficient homes (D, E, FG) exhibit actual energy use lower than theoretical, with an average difference ranging from 24% for D-rated homes to 56% for F- and G-rated homes. There are also significant differences by dwelling type. Apartments and detached dwellings feature a deficit in the region of 30%. Semi-detached homes semi-detached (19%) and terrace houses (7%) feature a smaller deficit.

A test of equality of medians (Snedecor & Cochran, 1989) reports the same level of significance and direction of results as the *t*-test of means. Table 7 shows a greater-than-theoretical energy use for efficient homes and lower-than-theoretical energy use for less efficient homes, a finding which is consistent with other estimates of the EPG (Cozza et al., 2020; Majcen et al., 2013; van den Brom et al., 2018). However, this result has not previously been shown using a measure of whole-home energy use. In particular, Cozza et al. (2020) find a median EPG of − 11% and mean EPG of − 6% for a sample of Swiss dwellings. In this study, the median EPG is similar (10.8%), but the mean difference is far greater (− 17%). This confirms the presence of an EPG in the Irish context but suggests that the EPG may be larger.

Results using a measure of the dependent variable that features an absolute appliance deduction still suggests a minor difference 677 kWh/year between the highest and lowest actual energy use averages (Table 8). Within EPC bands, results suggest that an EPG exists. The overall average difference is smaller (1105 kWh, − 8.40%), yet larger positive differences are observed for AB-rated homes (53.61%). C-rated homes also consume more than the theoretical amount (11.20%). FG-rated homes consume less than theoretical (− 51.28%), but the magnitude of this difference is smaller. A test of equality of medians is showing similar significance of results.

**Table 8:** Difference between annual actual (AQ) and theoretical (TQ) energy use (AA_Absolute_)

		Actual annual energy use (AQ)		Theoretical annual energy use (TQ)		*t*-test of equality of means			
	*n*	Mean AQ	Median AQ	Mean TQ	Median TQ	Difference		SE	*P* value
						Mean	%		
AQ_All_ – TQ	19,251	12,044	11,158	13,148	11,402	− 1105	− 8.40	68	0***
*EPC Grade*									
AB	2601	11,630	10,499	7571	6620	4059	53.61	147	0***
C	8269	12,039	11,356	10,826	9734	1213	11.20	83	0***
D	4835	12,119	11,207	14,353	12,826	− 2234	− 15.57	120	0***
E	2051	12,307	11,490	18,133	16,300	− 5826	− 32.13	195	0***
FG	1495	12,187	10,773	24,962	22,466	− 12,800	− 51.28	308	0***
*Dwelling*									
Apartment	1674	8617	7507	11,595	10,983	− 2978	− 25.68	184	0***
Detached	2316	15,620	15,003	19,385	17,184	− 3765	− 19.42	267	0***
Semi-detached	6905	12,702	12,063	14,008	12,495	− 1306	− 9.32	111	0***
Terrace	8356	11,195	10,584	11,020	9490	175	1.59	93	0.06*

^***^P < 0.01, **P < 0.05, *P < 0.10. Note: Units in kWh/year. Sample features 9923 observations of 1 year of actual energy use and a further 9328 observations from the same sample of houses with a second year of actual energy use. Medians reported. A test of equality of medians (using signtest STATA command (Snedecor & Cochran, 1989)) confirms the same significant differences exist as the *t*-test of means (displayed above)

**Fig. 3:**
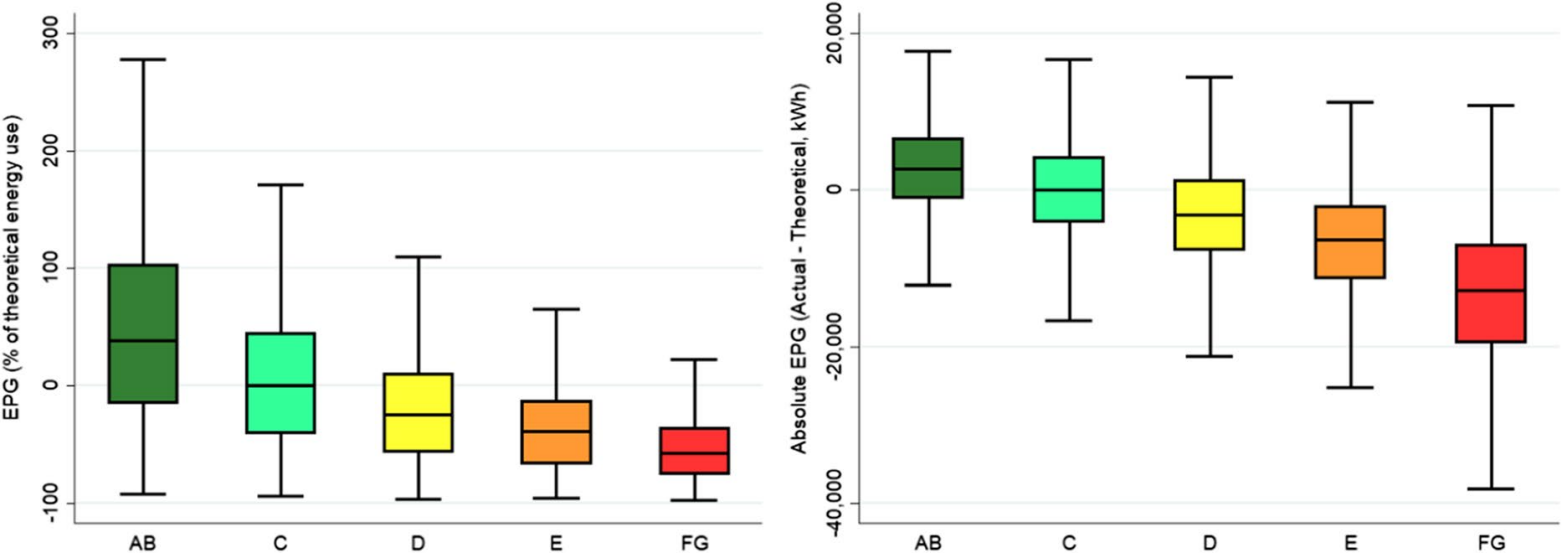
Comparison of Energy Performance Gap. Note: the left panel presents the EPG as the difference between actual and theoretical energy use as a percentage of theoretical energy use. The right panel presents the EPG in absolute differences. Each box reflects the interquartile range of EPG, with whiskers denoting the adjacent value. Sample includes 19,251 observations with 9923 observations of 1 year of actual energy use and a further 9328 observations featuring a second year of observed actual energy use. Figure reflects the relative appliance adjustment

Figure 3 illustrates the extent of the EPG across the spectrum of dwelling energy efficiency. The left panel features the EPG as a percentage of the theoretical EPC. The right panel features the EPG in absolute terms. It emphasises the substantial differences in the EPG, with a positive EPG for the most efficient dwellings and a negative EPG for the least efficient dwellings.

### Bimonthly results

Section Annual results provided evidence of an EPG across the entire EPC spectrum on an annual basis. Sections Bimonthly results and Bimonthly results: Split by EPC investigate the factors associated with actual energy use at the bimonthly frequency to better understand seasonal differences (Tables 9, 10, 11 and 12), using a linear regression (Eqs. 2 and 3). There are 149,518 data points in these regressions, indicated in the fourth row from the bottom in each table. In addition to the EPC and dwelling type, each regression controls for the following dwelling characteristics that are related to dwelling energy use, obtained from the EPC: number of floors, year of construction, percentage of home classed as living area (from Section Dwelling Characteristics and Weather). Regressions also control for weather using a measure of heating degree days and bimonthly rainfall.

**Table 9:** Bimonthly results — continuous EPC (AA_Relative_)

Dep Var: AQ actual energy use (kWh/bimonth)	Model 1		Model 2		Model 3	
	Coef	SE	Coef	SE	Coef	SE
TQ (kWh/bimonth)	0.466^***^	(0.042)	0.202^***^	(0.063)	0.222^***^	(0.061)
EPC = AB (REF)						
C	− 33.080	(67.636)	63.796	(69.185)	67.532	(66.217)
D	− 50.590	(72.906)	132.875^*^	(76.916)	131.218^*^	(73.388)
E	− 204.105^**^	(94.404)	14.525	(99.869)	32.140	(95.389)
FG	35.592	(117.020)	109.705	(113.436)	71.718	(111.735)
TQ#AB (REF)						
TQ#C	− 0.100^**^	(0.048)	− 0.027	(0.049)	− 0.041	(0.047)
TQ#D	− 0.183^***^	(0.047)	− 0.071	(0.051)	− 0.094^*^	(0.049)
TQ#E	− 0.185^***^	(0.050)	− 0.043	(0.057)	− 0.076	(0.055)
TQ#FG	− 0.321^***^	(0.049)	− 0.102^*^	(0.059)	− 0.124^**^	(0.057)
Detached (REF)						
Apartment			5.487	(41.743)	− 56.685	(39.239)
Semi-detached			− 17.547	(32.802)	− 47.427	(31.008)
Terrace			− 18.061	(35.134)	− 53.857	(33.098)
1 Floor (REF)						
2 Floors			518.289^***^	(33.150)	492.860^***^	(31.563)
3 Floors			829.710^***^	(46.477)	784.223^***^	(44.264)
4 Floors			552.210^***^	(206.635)	483.133^**^	(193.952)
Floor area (m_2_)			8.480^***^	(1.297)	7.806^***^	(1.235)
Year of construction			− 0.040	(0.366)	− 0.052	(0.344)
Living area percent			− 2.032^*^	(1.103)	− 3.294^***^	(1.055)
Heating degree days			27.651^***^	(0.313)		
Total precipitation (cm)			23.491^***^	(0.674)		
Bimonthly Time Dummy					Yes	Yes
Constant	1024.657^***^	(53.899)	− 1425.67^*^	(751.461)	1857.671^***^	(707.585)
N	149,518		149,518		149,518	
r2	0.028		0.112		0.255	
AIC	2,634,556		2,621,070		2,594,909	
BIC	2,634,655		2,621,278		2,595,246	

Asterisks note significance at the 10 percent (*), 5 percent (**), or 1 percent (***) level. Standard errors in brackets. Model 1 features a significant interaction for the continuous and categorical EPC independent variables

**Table 10:** Bimonthly results — continuous EPC (AA_Absolute_)

Dep Var: AQ actual energy use (kWh/bimonth)	Model 1_Abs_		Model 2 _Abs_		Model 3 _Abs_	
	Coef	SE	Coef	SE	Coef	SE
TQ (kWh/bimonth)	0.586^***^	(0.052)	0.257^***^	(0.077)	0.282^***^	(0.074)
EPC = AB (REF)						
C	− 36.686	(83.897)	80.930	(85.970)	85.474	(82.242)
D	− 50.335	(90.273)	172.676^*^	(95.467)	170.472^*^	(91.035)
E	− 234.916^**^	(117.127)	27.987	(124.325)	49.941	(118.660)
FG	69.487	(141.041)	154.345	(137.354)	106.771	(134.915)
TQ#AB (REF)						
TQ#C	− 0.126^**^	(0.060)	− 0.035	(0.060)	− 0.051	(0.058)
TQ#D	− 0.232^***^	(0.058)	− 0.091	(0.063)	− 0.120^**^	(0.061)
TQ#E	− 0.234^***^	(0.062)	− 0.055	(0.070)	− 0.096	(0.067)
TQ#FG	− 0.407^***^	(0.061)	− 0.134^*^	(0.073)	− 0.162^**^	(0.070)
Detached (REF)						
Apartment			1.912	(51.753)	− 75.859	(48.617)
Semi-detached			− 26.190	(40.772)	− 63.580^*^	(38.530)
Terrace			− 28.773	(43.670)	− 73.615^*^	(41.127)
1 Floor (REF)						
2 Floors			640.371^***^	(40.979)	608.591^***^	(38.963)
3 Floors			1031.274^***^	(57.534)	974.431^***^	(54.734)
4 Floors			677.805^***^	(255.956)	591.653^**^	(240.308)
Floor area (m_2_)			10.473^***^	(1.584)	9.632^***^	(1.504)
Year of construction			− 0.180	(0.454)	− 0.195	(0.426)
Living area percent			− 2.413^*^	(1.368)	− 3.990^***^	(1.307)
Heating degree days			34.441^***^	(0.390)		
Total precipitation (cm)			29.411^***^	(0.840)		
Bimonthly time					Yes	Yes
Constant	1009.958^***^	(66.653)	− 1770.902^*^	(933.531)	2329.161^***^	(878.001)
N	149,518		149,518		149,518	
r2	0.029		0.113		0.257	
AIC	2,700,145		2,686,646		2,660,127	
BIC	2,700,244		2,686,855		2,660,464	

Asterisks note significance at the 10 percent (*), 5 percent (**), or 1 percent (***) level. Standard errors in brackets. Model 1 features a significant interaction for the continuous and categorical EPC independent variables

**Table 11:** Bimonthly results — continuous EPC, by EPC category (AA_Relative_)

Dep Var: AQ actual energy use (kWh/bimonth)	Model 4 — EPC label				
	AB	C	D	E	FG
TQ (kWh/bimonth)	0.21^**^	0.16^**^	0.10	0.44^***^	0.09^*^
	(0.09)	(0.06)	(0.08)	(0.13)	(0.04)
Detached (REF)					
Apartment	31.98	− 82.10	− 0.66	− 57.27	− 179.64
	(101.10)	(62.65)	(83.19)	(136.72)	(166.66)
Semi-detached	− 97.60	− 110.07^**^	27.47	− 48.44	117.17
	(79.59)	(46.16)	(61.41)	(111.91)	(116.53)
Terrace	− 33.00	− 76.90	− 16.62	− 53.52	− 57.04
	(81.51)	(50.51)	(66.16)	(115.57)	(119.99)
1 Floor (REF)					
2 Floor	357.15^***^	500.60^***^	598.68^***^	426.30^***^	441.32^***^
	(114.05)	(51.35)	(54.03)	(87.08)	(100.78)
3 Floor	673.08^***^	819.08^***^	725.50^***^	771.94^***^	708.54^***^
	(133.47)	(67.14)	(91.89)	(146.46)	(189.89)
4 Floors	1110.28^***^	357.92	551.62^*^		
	(149.40)	(246.11)	(329.90)		
Floor area (m_2_)	7.45^***^	8.41^***^	9.84^***^	− 9.41	8.32^**^
	(2.05)	(1.98)	(3.65)	(7.38)	(3.87)
Year built	− 1.38	− 0.58	0.01	2.81^***^	− 1.94^*^
	(0.95)	(0.59)	(0.66)	(0.84)	(1.11)
Living area percent	− 12.06^***^	− 3.86^**^	0.51	− 3.93	− 0.26
	(2.98)	(1.69)	(1.92)	(2.86)	(3.97)
Bimonthly Time	Yes	Yes	Yes	Yes	Yes
Constant	3333.49^*^	2764.72^**^	1605.77	-3851.8^**^	5548.46^***^
	(2011.66)	(1184.99)	(1334.01)	(1658.07)	(2126.24)
N	20,116	64,456	37,506	15,897	11,543
r2	0.273	0.260	0.255	0.265	0.219
AIC	346,797	1,113,643	651,925	277,468	203,840
BIC	346,995	1,113,879	652,147	277,659	204,023

Asterisks note significance at the 10 percent (*), 5 percent (**), or 1 percent (***) level. Standard errors in brackets

**Table 12:** Bimonthly results — continuous EPC, by EPC category (AA_Absolute_)

Dep Var: AQ actual energy use (kWh/bimonth)	Model 4_Abs_ — EPC Label				
	AB	C	D	E	FG
EPC (kWh/bimonth)	0.28^***^	0.20^**^	0.13	0.56^***^	0.09^*^
	(0.10)	(0.08)	(0.10)	(0.16)	(0.05)
Detached (REF)					
Apartment	46.28	− 107.23	− 9.15	− 81.08	− 238.90
	(125.04)	(78.08)	(103.02)	(170.65)	(199.12)
Semi-detached	− 120.69	− 140.87^**^	30.48	− 69.20	131.12
	(98.38)	(57.53)	(76.44)	(139.03)	(142.58)
Terrace	− 38.88	− 102.54	− 25.43	− 80.06	− 84.14
	(101.16)	(62.95)	(82.25)	(143.65)	(146.65)
1 Floor (REF)					
2 Floor	447.60^***^	617.86^***^	735.36^***^	528.56^***^	550.79^***^
	(139.06)	(63.87)	(66.94)	(107.96)	(121.96)
3 Floor	840.97^***^	1016.06^***^	903.99^***^	963.06^***^	889.66^***^
	(162.93)	(83.37)	(114.67)	(181.54)	(230.83)
4 Floors	1383.02^***^	440.42	664.84		
	(183.26)	(305.80)	(409.67)		
Floor area (m_2_)	8.76^***^	10.44^***^	12.12^***^	− 12.54	11.88^**^
	(2.48)	(2.47)	(4.54)	(9.17)	(4.65)
Year built	− 1.74	− 0.86	− 0.14	3.31^***^	− 2.47^*^
	(1.18)	(0.73)	(0.82)	(1.04)	(1.35)
Living area percent	− 14.99^***^	− 4.75^**^	0.65	− 4.52	0.35
	(3.66)	(2.11)	(2.38)	(3.54)	(4.85)
Bimonthly Time	Yes	Yes	Yes	Yes	Yes
Constant	5796.75^**^	3779.70^**^	2025.20	− 4260.4^**^	6792.98^**^
	(2473.12)	(1479.84)	(1658.99)	(2108.26)	(2681.35)
N	20,116	64,456	37,506	15,897	11,543
r2	0.276	0.262	0.257	0.267	0.223
AIC	355,463	1,141,976	668,373	284,384	208,743
BIC	355,660	1,142,212	668,595	284,575	208,927

Asterisks note significance at the 10 percent (*), 5 percent (**), or 1 percent (***) level. Standard errors in brackets

Actual bimonthly energy use (${\mathrm{AQ}}_{\mathrm{t}}$) is modelled as a function of the bimonthly theoretical energy use (${\mathrm{TQ}}_{\mathrm{t}}=\mathrm{TQ}/6$), a full interaction with the categorical EPC, specific dwelling characteristics and weather controls (Table 9). Standard errors are clustered at the household level. Table 9 shows a less than 1:1 relationship between changes in actual and theoretical energy use. Model 1 suggests that, on average, a 1 kWh/bimonth increase in theoretical energy use leads to a 0.47 kWh increase in actual bimonthly energy use. In Model 2 the theoretical EPC coefficient falls to 0.198 and features significant effects for dwelling size and weather covariates. A one square metre increase in dwelling size is associated with 8.69 kWh higher actual energy use, on average. Relative to a one floor dwelling, houses with a second floor, on average, use 548kWh more each period. Results suggest that larger homes consume more energy and that actual energy use rises during colder or periods with more rain.

Model 3 replaces the weather variables with a categorical time variable. Relative to November–December 2014, we observe lower actual use in spring/summer and higher energy use in autumn/winter. The coefficients for the EPC and dwelling features are largely unchanged. Models 1–3 also interact the continuous EPC with its categorical form. Significant interactions in Model 1 suggest a heterogenous relationship between the continuous EPC and actual energy use, depending on the theoretical level of energy efficiency. Following AIC and BIC criteria, we consider Model 3 the most appropriate.^16^

Results in Table 10 are obtained using the variant of actual energy use that features an absolute reduction in appliance use (AA_Absolute_) are in line with those in Table 9. The exception is that every significant coefficient is larger in magnitude than before, reflecting a stronger association between theoretical energy use, dwelling characteristics and actual energy use.

### Bimonthly results: split by EPC

This section answers the third research question, which investigates whether the effects from Model 3 are heterogeneous across the levels of the EPC. These results are presented in Model 4, which splits the sample according to EPC (Table 11). Results show decreasing explanatory power for the least energy efficient dwellings. This finding is similar to van den Brom et al. (2018), who find their EPC to be more reliable for efficient households.

Differences are observed in the magnitude of the coefficient for the continuous measure of theoretical energy use (EPC). Average effects range from 0.16 to 0.45, which differs from the same coefficient in Model 3 (0.22). When split by EPC, dwelling type is only significantly lower for C-rated apartments and semi-detached houses. There are significant effects for dwelling size throughout and a significant floor area effect in all except E-rated homes, with larger average effects (8.15–9.78) than in the pooled Model 3 (8.13).

Using the alternative version of our dependent variable (absolute appliance adjustment), we see similar results (Table 12). Average effects range from 0.20 to 0.56, suggesting that changes in theoretical energy use are more closely related to changes in actual energy use for the most energy efficient dwellings. Although results at the bimonthly level do not prove the existence of an EPG (Section Annual results), they highlight the statistically significant role of the EPC, dwelling characteristics and seasonality when modelling actual energy use.

## Discussion

The key insight from this study is the striking lack of variation in average actual energy use across the sample (457 kWh/year). This suggests that occupant demand for energy may not be as responsive to dwelling energy efficiency, which has been observed in the energy use of commercial buildings (Better Buildings Partnership 2019). This study also finds evidence of an Energy Performance Gap (EPG) for the Irish EPC, with significant differences between actual and theoretical energy use. Annual actual energy use is below the theoretical level, with a mean deficit of 2279 kWh/year (− 17%) and a median deficit of 1235 kWh/year (10.83%). By comparison, Cozza et al. (2020) find a median EPG of − 11% and mean EPG of − 6% for a sample of Swiss dwellings. In this study, the median EPG is similar (10.8%), but the mean difference is far greater (− 17%). This confirms the presence of an EPG in the Irish context but suggests that the EPG may be larger.

The size of the EPG varies by the level of the EPC. For the most efficient homes, actual energy use exceeds theoretical, with an average difference in the range of 39.6% for AB-rated homes. For the least efficient homes, actual energy use is below theoretical, with an average deficit of 24% for D-rated homes, 39% for E-rated homes and 56% for FG-rated homes.

These results have significant policy implications, as a nationwide upgrade of dwelling energy efficiency may be ineffective or counter-productive, with under-heating in the least efficient homes potentially leading to over-heating post-upgrade. This is an important consideration, as it may run counter to policy targets of reducing energy use (while accepting it would likely improve occupant comfort and welfare). This is especially relevant considering the fact that the Energy Performance Gap is widely accepted in the research community, but often ignored in policy discourse (Gram-Hanssen & Georg, 2018).

Findings are in line with similar studies of the EPG (Cozza et al. 2020; Majcen et al. 2013; van den Brom et al. 2018). Similar to the observation of Delghust et al. (2015), this study emphasises the importance of accounting for electricity use, instead of limiting the focus strictly space and water heating (Scheer et al. 2013). This is especially important as homes become increasingly energy efficient and electricity reliant.

The lack of variation in energy use across dwelling of very different theoretical efficiency presents opportunities for research across households of differing dwelling energy efficiency and socioeconomic status. For example, it could be the case that occupants of energy efficient homes have paid a premium for a home that can be heated at a lower effective per-unit cost. Similarly, possible explanations for the under-heating observed in energy inefficient homes could be due to other barriers to energy use such as fuel poverty, which have been established in research elsewhere (Coyne et al. 2018).

Results at the bimonthly frequency indicate that a 1 kWh increase in bimonthly theoretical energy use is associated with a 0.222 kWh increase in actual energy usage, on average. Other results suggest the EPC broadly works as intended, with a less efficient EPC being associated with greater actual energy use, when also controlling for key dwelling characteristics and seasonality. The coefficient values for dwelling floor size (7.81 kWh/bimonth) indicate that larger homes tend to consume more energy. When split by EPC category, greater explanatory power for more efficient homes is observed. The model better explains variation for more efficient homes, suggesting there is greater uncertainty when modelling less efficient homes, a finding that is consistent with van den Brom et al. (2018).

Future work might seek to address some of the limitations of this study. One concern is that the actual energy data observed is understated if a home uses another fuel source, e.g. open wood burning stove. This is true of many studies that focus on one space heating fuel. In this study, this risk is minimized by focusing homes with either natural gas or electricity as their heating fuel. To address sample attrition that may arise from customers switching energy provider,^17^ criteria based on the number of readings, the level of missingness and for unrealistically low metered energy use is applied to ensure sufficient energy use is observed (see Appendix 2). Customer switching could be addressed with access to data from more utilities. The addition of household socioeconomic information could help to explain the main result of a lack of variation in actual energy use across the sample. Finally, future research could seek to quantify changes in whole-home energy use before and following a home energy retrofit. This would require a dataset with metered energy use and EPCs pre- and post-retrofit.

## Conclusion

This paper investigates the difference between theoretical energy use denoted by a residential Energy Performance Certificate (EPC) with actual energy use for a sample of 9923 households in Ireland from late 2014 to mid-2017. It is the first paper to test for the presence of an Energy Performance Gap using a measure of whole-home energy use for a non-social housing sample of dwellings that do not receive a retrofit. It focuses on homes heated by natural gas and electricity to profile whole-home energy use and capture fuel switching. Households that underwent a retrofit during the observed period are excluded from the sample in order to isolate the difference in actual energy use and the theoretical level created by the engineering-based model that informs the EPC.

Results show there is very little difference in actual average consumption for households across the EPC spectrum. There is a less than five percent discrepancy (457 kWh/year) between the highest and lowest average value. This is a surprising observation which warrants further investigation to understand the factors underlying this result. Analysis within EPC bands shows evidence of an Energy Performance Gap (EPG), with lower-than-expected energy use for houses with low energy efficiency and higher-than-expected energy use for energy efficient houses. For more efficient homes (AB, C) the average difference ranges from + 39% to − 56% of the relevant EPC value. Less efficient homes (E, FG) feature actual energy use lower than predicted, with an average difference ranging from − 23 to − 56% below the relevant EPC. Results using a measure of actual energy use with an absolute deduction for appliance usage (instead of relative) display similar results.

These findings are in line with similar studies of the EPG that focused exclusively on social housing tenants (Majcen et al., 2013; van den Brom et al., 2018). Additional results show a heterogenous relationship between theoretical energy efficiency and actual energy use across EPC levels. This is consistent with prior work that found the EPC has less explanatory power for the least efficient homes (Cozza et al., 2020; Sunikka-blank & Galvin, 2012; van den Brom et al., 2018) and the ‘prebound’ effect (Cozza et al., 2020; Sunikka-blank & Galvin, 2012).

Policymakers could seek to improve the EPC by including historical energy use information. This could be facilitated by the upcoming rollout of residential smart meters as part of the Climate Action Plan (Government of Ireland, 2019). Since the Irish EPC is consistent with EU guidance, it is likely that the issues identified in this paper could be present in other contexts, especially in the UK, as the Irish EPC is based on the UK Standard Assessment Procedure for dwelling energy ratings (SEAI 2012) and a similar lack of variation in actual energy use has been observed in commercial buildings (Better Buildings Partnership 2019).

Future work could include additional utilities, socioeconomic information and track occupants over time (as is done in a retrofit study by Aydin et al. (2017)) to minimise customer attrition and include additional relevant covariates. This would enable researchers to understand the factors causing differences in the most and least efficient homes.

## Appendix 1: Constructing a comparable measure of actual energy use

**Fig. 4:**
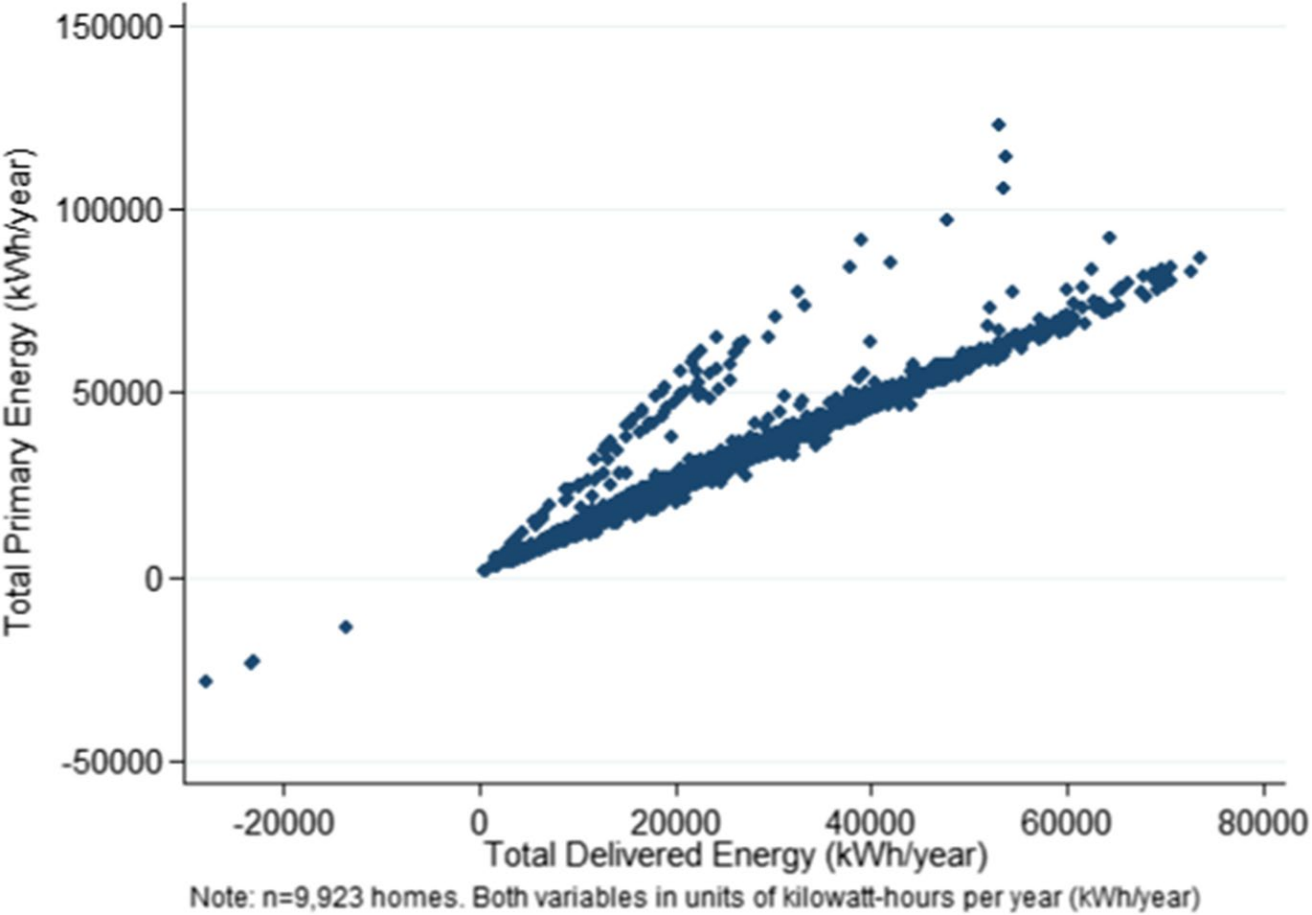
Primary v delivered energy

This paper tests for differences in actual energy use (AQ) with the theoretical level from the Irish residential EPC (TQ). Whenever actual energy use is mentioned (Eq. 6) is applies to the variable created that is comparable to theoretical energy use (Eq. 7). Actual meter readings are aggregated, weighted by the heatable floor area (per EPC) and account for the difference between ‘primary’ and ‘delivered’ energy use (per EPC). The actual energy use variable must be adjusted to reflect ‘primary’ energy consumed. Per SEAI, ‘primary’ energy use includes the energy consumed in the house plus an overhead for energy used in its generation and transmission. ‘Delivered’ energy is only what is consumed within the home. In the SEAI database of 729,609 homes, ‘primary’ is 39% larger than ‘delivered’ on average. In the sample of 9923 homes, ‘primary’ is 22% larger than ‘delivered’ on average (Fig. 4). We then adjust it to only reflect energy for space and water heating, lighting and ventilation.

6 $$\mathbf{EPC}:\quad\quad {\mathrm{TQ}}_{\mathrm{i}}=\frac{{\mathrm{PrimaryEnery}}_{\mathrm{i}}}{{\mathrm{HeatableFloorArea}}_{\mathrm{i}}}$$

7 $$\mathbf{A}\mathbf{c}\mathbf{t}\mathbf{u}\mathbf{a}\mathbf{l}\mathbf{E}\mathbf{n}\mathbf{e}\mathbf{r}\mathbf{g}\mathbf{y}\mathbf{U}\mathbf{s}\mathbf{e}: {\mathrm{AQ}}_{\mathrm{it}}=\frac{{\sum \mathrm{Meter readings}}_{\mathrm{it}}}{{\mathrm{HeatableFloorArea}}_{\mathrm{i}}} * \frac{{\mathrm{TotalPrimaryEnergy}}_{\mathrm{i}}}{{\mathrm{TotalDeliveredEnergy}}_{\mathrm{i}}}*{\mathrm{AA}}_{j}$$

Fig. 4

The EPC is based on heatable floor area (in units of kWh/m^2^/year). Heatable and total floor area variables are present in the SEAI data. One source of sample attrition is that approximately 11% of dwellings in the SEAI database report a heatable floor area of zero, the majority being mid-floor apartments where heat loss is through the external wall (Table 13). These are excluded as they prevent the actual energy use variable from being constructed.

**Table 13:** Homes with zero heatable floor area

	Apartment	Basement	Detached	Ground apartment	Maisonette	Mid floor apartment	Top floor apartment	Total
Houses	2,359	2	1	815	2357	42,276	33,406	81,216

Source: SEAI BER database (*n* = 729,609)

**Fig. 5:**
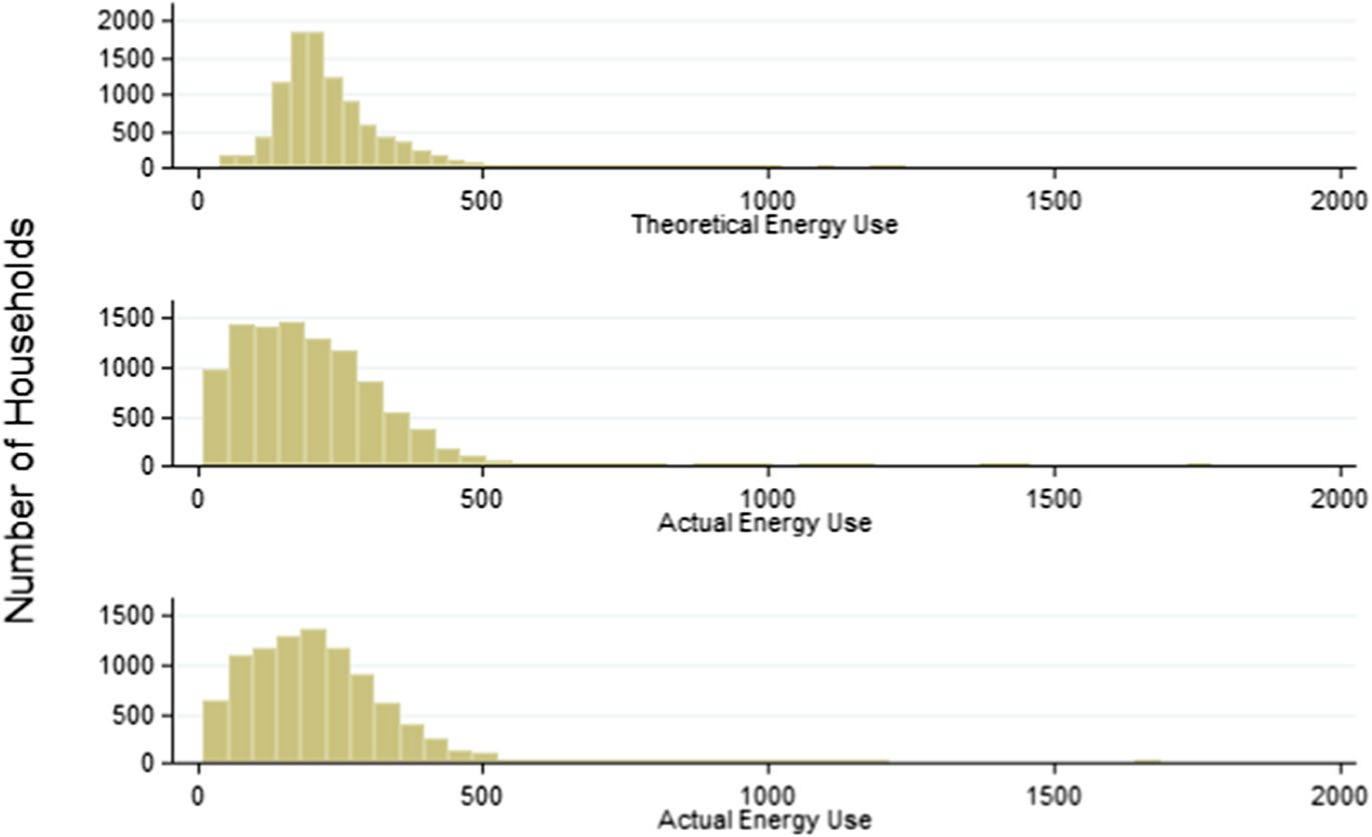
Distributions of continuous theoretical v actual energy use (Y1, Y2)

Figure 5 compares the distribution of the continuous theoretical energy use (TQ) with the actual energy use (AQ) for the first and second full years observed (Y1, Y2). The distributions are within a similar range, which suggests that the removal of extreme values was successful.

Fig. 5

## Appendix 2: Data cleaning process

This section details the data cleaning process in this paper. We describe the linking process, discuss how energy data are merged and list the data cleaning and exclusion criteria used.

1. **Linking customers**
  - Gas fuelled homes are identified by linking Electric Ireland gas customer accounts with the corresponding Electric Ireland electricity account.
  - Electricity accounts are anonymously merged with the SEAI dwelling data using the electric meter number (MPRN), which was unobservable to the research team.
  - There are 286,523 unique customer matches between the original Electric Ireland measured energy use data and the SEAI dwelling data. Of this, 21,198 are unique matches for a gas customer account that is linked to an electricity customer account.
2. **Energy data merge and sample restrictions**

The original energy dataset features 30,045,696 daily energy readings (28,563,625 electricity, 1,482,071 gas) beginning November 2011. We drop households with no match in the SEAI dwelling data. Readings are aggregated bimonthly and adjusted to reflect the period of use, e.g. A reading in March 2015 reflects usage in January 2015. Additional observations are dropped for the following reasons:

- Total household metered energy usage is zero.
- House is not heated by gas (per SEAI).
- Multiple meters for a house (per SEAI).
- A house received a grant-supported retrofit during the observed period (per SEAI).
- Drop electricity readings before the start of the gas sample (November 2014) to focus on the common period of electricity and gas use.
- A ratio of the number of missing periods to the number of periods present is created. This ratio is equal to 0.75 if a household is present for 16 periods but if missing for any four periods. Any household with a ratio less than 0.5 is dropped, which does not discriminate against homes that enter the data later.
- Drop any household with a gap between observations of at least 6 months. Although the customer might be present during the entire sample period, such a large missing period makes it unsuitable for analysis, especially for annual values.
- Drop any house with fewer than six observations (a full year of readings).
- Drop any house with an annual energy usage value (Y1, Y2) reading in the top or bottom one percent of the distribution to observe households with realistic energy use.
- Drop homes with an SEAI heatable floor area of less than 10m^2^. Mostly apartments.
- Remove households with a delivered energy value in the top or bottom 1% of the distribution. As noted in Appendix 1, the SEAI dataset includes two variables of calculated annual use, one reflecting consumed energy (delivered energy) and the other including an overhead for energy generation (primary energy).

## Appendix 3: Weather and energy price controls

Fig. 6

**Fig. 6:**
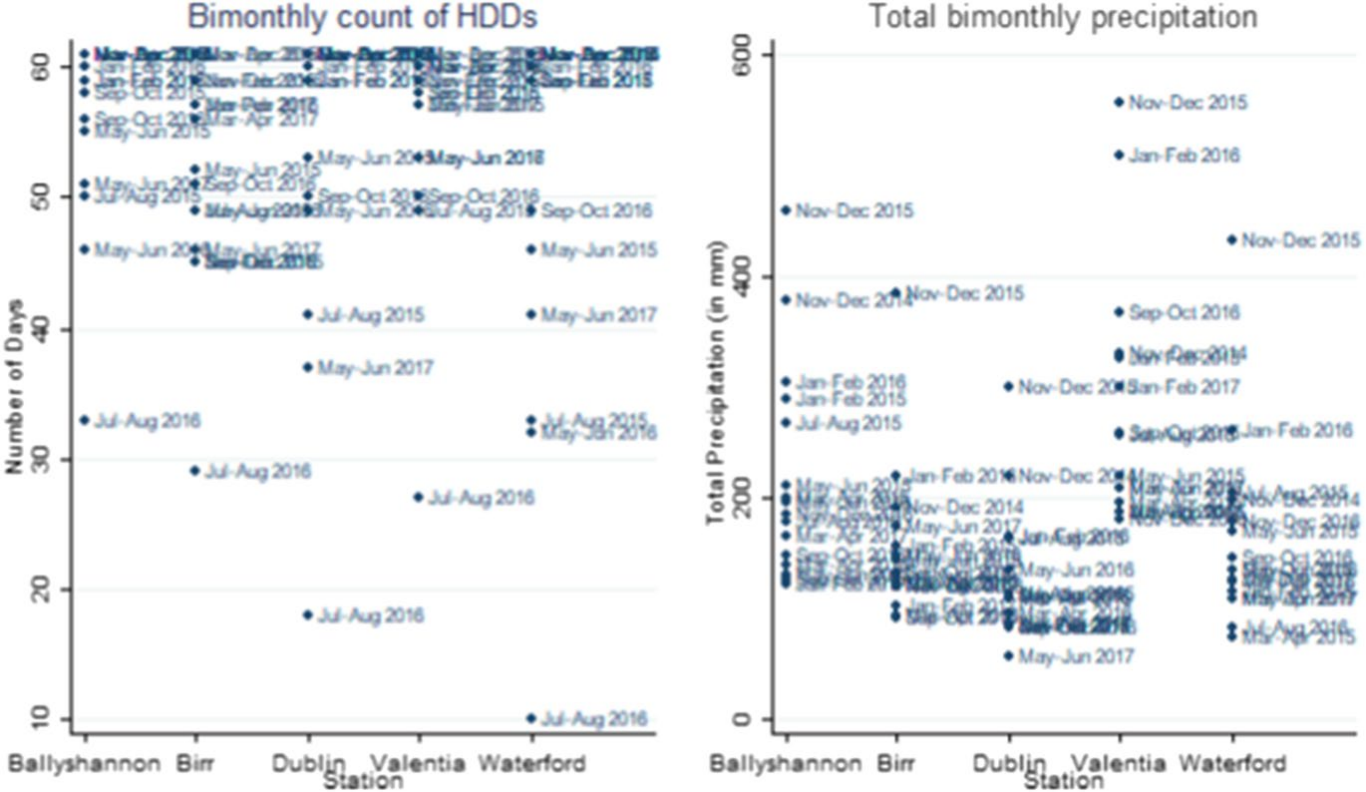
Bimonthly heating degree days and rainfall (by station). Source: Met Eireann. Note: A Heating Degree Day occurs when the mean temperature falls below 15.5 °C

**Fig. 7:**
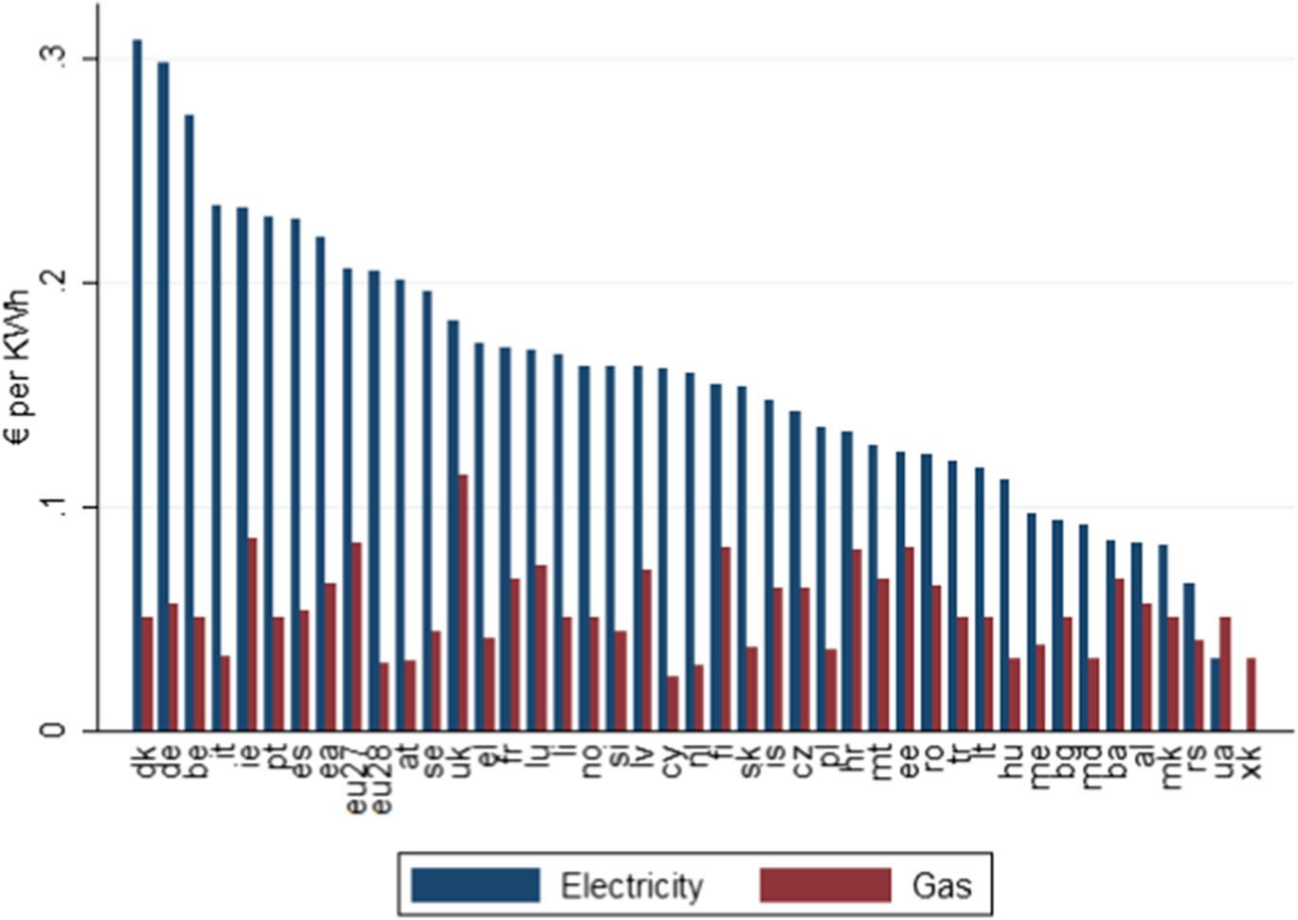
EU 2016 H2 electricity and gas price. Source: Eurostat H2 2016 Energy Prices

Fig. 7

## Appendix 4: The Irish regulator annual energy use benchmark

The Irish Commission for Regulation of Utilities (CRU) provides reference values for annual domestic energy use to be used by price comparison websites and energy providers. 2017 annual averages were set at 4200 kWh and 11,000 kWh for electricity and natural gas, respectively. Tables 14 and 15 shows the CRU annual means split by electricity meter type and dwelling type for gas. The CRU observes average electricity use by tariff, with urban and rural 24 h tariffs below the national average. However, urban and rural day/night tariffs show an average above the national mean. The CRU gas data shows that the national average of 11,000 is higher than what would be expected for an apartment. Unfortunately, we cannot control for tariff type in our data. This underscores the need to consider appropriate reference points for annual national averages when considering our constructed variable of actual energy use and how it might vary by tariff and property type.

**Table 14:** Assumptions regarding appliance-specific electricity energy use (for constructing AA_Absolute_)

	Fridge-freezer	Oven	Microwave	Electric kettle	Toaster	Washing machine^1^	Vacuum	LCD TV	Total
Annual consumption (kWh)	427	290	56	167	22	178	18	199	1357

Source: Owen & Foreman (2012). ^1.^Values assume a multiple person household. The annual average lighting energy consumption of 548 kWh is disregarded in our analysis as the EPC accounts for lighting

**Table 15:** CRU average energy consumption

	Annual average electricity consumption (kWh)		Annual average gas consumption (kWh)
CRU mean (2017)	4200		11,000
*Electricity tariff type*		*Gas dwelling type*	
Urban 24 h	3600 (− 14%)	Apartment (1–3 bed)	7000 (− 46%)
Urban day/night tariff	6200 (+ 48%)	House (1–3 bed)	10,500 (− 5%)
Rural 24 h	3900 (− 7%)	Large house (4–6 bed)	13,000 (+ 18%)
Rural day/night tariff	12,000 (+ 286%)	Standalone residential	15,000 (+ 36%)

Source: CRU decision paper CER/17042

## Appendix 5: Robustness check 1 — annual results (split by year of energy use)

This section provides a robustness check on the earlier test of significant differences between annual values of theoretical and actual energy use. The body of the paper aggregates 19,251 observations of actual annual energy use. Here we split the sample into 9923 household-level observations for 1 full year of energy use and a further 9328 observations of a second full year of energy use. Comparing Tables 16 and 17 we observe a similar trend in differences between actual and theoretical energy use. Results for the second year of energy use feature a slightly smaller deficit between actual and theoretical energy use, with smaller deficits observed across every level of the EPC and property type. The only exception is the most energy efficient homes, with a slightly larger deficit for AB- and C-rated homes.

**Table 16:** Year 1: difference between annual actual (AQ) and theoretical (TQ) energy use (AA_Relative_)

	*n*	Mean AQ_Y1_	Mean EPC	Difference	% Difference	SE	*P* value
AQ_All,Y1_ – TQ	9923	10,532	13,152	− 2620	− 19.92%	86	0***
*EPC grade*							
AB	1351	10,392	7509	2882	38.38%	171	0***
C	4257	10,521	10,842	− 322	− 2.97%	100	0.002***
D	2486	10,546	14,373	− 3827	− 26.62%	147	0***
E	1059	10,745	18,159	− 7414	− 40.83%	246	0***
FG	770	10,499	24,990	− 14,500	− 58.02%	400	0***
*Dwelling type*							
Apartment	873	7,914	11,625	− 3711	− 31.92%	227	0***
Detached	1197	13,261	19,323	− 6061	− 31.37%	346	0***
Semi-detached	3565	11,026	13,996	− 2970	− 21.22%	140	0***
Terrace	4288	9,892	11,039	− 1146	− 10.38%	116	0***

^***^P < 0.01, **P < 0.05, *P < 0.10. Sample of 9923 homes with 9923 observations of 1 year of actual energy use and a further 9328 observations for the same houses with a second year of observed actual energy use

**Table 17:** Year 2: difference between annual actual (AQ) and theoretical (TQ) energy use (AA_Relative_)

	*n*	Mean AQ_Y2_	Mean EPC	Difference	% Difference	SE	*P* value
AQ_All,Y2_ – TQ	9328	11,229	13,144	− 1916	− 14.57%	87	0***
*EPC grade*							
AB	1250	10,761	7637	3124	40.90%	175	0***
C	4012	11,262	10,808	453	4.20%	99	0***
D	2349	11,309	14,332	− 3022	− 21.09%	147	0.6
E	992	11,326	18,105	− 6778	− 37.44%	242	0***
FG	725	11,458	24,932	− 13,500	− 54.15%	421	0***
*Dwelling type*							
Apartment	801	8333	11,563	− 3230	− 27.93%	233	0.002***
Detached	1119	14,194	19,451	− 5258	− 27.03%	354	0.181
Semi-detached	3340	11,796	14,021	− 2226	− 15.87%	139	0***
Terrace	4068	10,518	11,000	− 483	− 4.39%	117	0***

^***^P < 0.01, **P < 0.05, *P < 0.10. Sample of 9923 homes with 9923 observations of 1 year of actual energy use and a further 9328 observations for the same houses with a second year of observed actual energy use

## Appendix 6: Robustness check 2 — annual results (split by subsample)

As noted in the body of the paper, the sample of 9923 houses (149,518 readings) is divided across 8311 houses (124,763 readings) that never receive a retrofit and a further 1612 houses (24,755) that completed a retrofit prior to the start of our observed period of energy use. Results in the body of the paper report values for the entire sample, being explicit in how the sample excludes houses that change their dwelling energy efficiency over time. This appendix replicates those results, split by subsample, as a robustness check, to show no major discrepancy exists between houses in the sample (Tables 18 and 19).

**Table 18:** Never retrofit — difference between annual actual (AQ) and theoretical (TQ) energy use (AA_Relative_)

	*n*	Mean AQ	Mean EPC	Difference	% Difference	SE	*P* value
AQ_Control_ – TQ	16,091	10,657	12,985	− 2327	− 17.92%	68	0***
EPC grade							
AB	2317	10,205	7087	3118	43.99%	129	0***
C	6590	10,604	10,346	258	2.49%	77	0.001***
D	3861	10,711	14,045	− 3335	− 23.74%	114	0***
E	1858	11,081	17,995	− 6914	− 38.42%	179	0***
FG	1465	10,937	25,035	− 14,100	− 56.32%	294	0***
Dwelling type							
Apartment	1622	8088	11,501	− 3413	− 29.68%	165	0***
Detached	1723	13,391	19,390	− 5998	− 30.94%	301	0***
Semi-detached	5485	11,207	14,041	− 2834	− 20.18%	113	0***
Terrace	7261	10,167	10,998	− 831	− 7.56%	90	0***

^***^P < 0.01, **P < 0.05, *P < 0.10. Sample of 8311 homes that never avail of a grant-supported retrofit. 8311 observations of 1 year of actual energy use and a further 7780 observations for the same houses with a second year of observed actual energy use

**Table 19:** Already retrofit — difference between annual actual (AQ) and theoretical (TQ) energy use (AA_Relative_)

	*n*	Mean AQ	Mean EPC	Difference	% Difference	SE	*P* value
AQ_Treated_ – TQ	3160	11,949	13,980	− 2031	− 14.52%	138	0***
***EPC Grade***							
AB	284	13,540	11,517	2023	17.57%	375	0***
C	1679	11,966	12,711	− 745	− 5.86%	171	0***
D	974	11,735	15,573	− 3838	− 24.65%	250	0***
E	193	10,495	19,456	− 8961	− 46.06%	626	0***
FG	30	12,293	21,387	− 9094	− 42.52%	1318	0***
***Dwelling Type***							
Apartment	52	8954	14,544	− 5590	− 38.44%	1043	0***
Detached	593	14,644	19,371	− 4728	− 24.41%	408	0***
Semi-detached	1420	12,135	13,879	− 1744	− 12.57%	194	0***
Terrace	1095	10,392	11,165	− 772	− 6.92%	196	0***

^***^P < 0.01, **P < 0.05, *P < 0.10. Sample of 1612 homes that avail of a grant-supported retrofit before the period of energy use. 1612 observations of 1 year of actual energy use and a further 1548 observations for the same houses with a second year of observed actual energy use

## Appendix 7: Robustness check 3 — bimonthly results using categorical EPC

In addition to the continuous version of the EPC, we model the categorical version of the EPC to observe associations (Table 20). This is performed in order to consider the average effect of a one unit increase in actual energy use for a change in the EPC. This is appealing if we believe that occupants are aware of their EPC but are inattentive to the specific unit value. These results are consistent with those in the body of the paper using a continuous EPC and confirm that less efficient EPCs are associated with increasing levels of actual energy use.

**Table 20:** Bimonthly OLS results — categorical EPC (AA_Relative_)

Dep Var: Bimonthly measured energy use (kWh)	Model 5		Model 6		Model 7	
	Coef	SE	Coef	SE	Coef	SE
EPC label = AB	REF		REF		REF	
C	37.644	(26.467)	152.072^***^	(25.291)	143.463^***^	(23.823)
D	36.961	(28.886)	224.829^***^	(29.235)	189.794^***^	(27.623)
E	54.256	(35.535)	277.004^***^	(37.420)	231.953^***^	(35.467)
FG	49.832	(41.052)	310.585^***^	(44.758)	241.323^***^	(42.668)
Detached (REF)						
Apartment			− 9.963	(41.907)	− 72.374^*^	(39.406)
Semi-detached			− 19.210	(32.817)	− 49.929	(31.025)
Terrace			− 26.649	(35.015)	− 63.286^*^	(32.978)
1 Floor (REF)						
2 Floors			491.419^***^	(32.992)	467.667^***^	(31.387)
3 Floors			794.670^***^	(46.279)	750.215^***^	(44.048)
4 Floors			508.383^**^	(201.707)	443.997^**^	(190.195)
Floor area (m_2_)			13.923^***^	(0.566)	13.342^***^	(0.540)
Year built			− 0.374	(0.358)	− 0.408	(0.336)
Living area percent			− 2.558^**^	(1.091)	− 3.708^***^	(1.044)
Total Heating Degree Days			27.656^***^	(0.313)		
Total rainfall (cm)			23.470^***^	(0.676)		
Bimonthly Time					Yes	Yes
Constant	1613.435^***^	(23.329)	− 805.289	(731.714)	2537.047^***^	(688.257)
N	149,518		149,518		149,518	
r2	0.000		0.111		0.254	
AIC	2,638,800		2,621,245		2,595,109	
BIC	2,638,849		2,621,404		2,595,396	

Asterisks note significance at the 10 percent (*), 5 percent (**), or 1 percent (***) level. Standard errors in brackets

## Appendix: Robustness check 4 — fuel-specific results

Results in the body of this paper consider a measure of actual energy use that includes electricity and gas, while deflating to account for appliance use. This is designed to be comparable to the EPC. Many other studies of the EPG focus on the comparison between gas energy use and the EPC (Aydin et al. 2017; Cozza et al. 2020; Sunikka-blank & Galvin, 2012). This section presents annual results that perform a *t*-test of means for gas. If this shows similar results to Section Annual results, this suggests that the evidence on the EPG observed is all due to a difference in gas consumption from the theoretical EPC level.

Table 21 highlights that the average EPG is 28% when only studying gas energy use. This is larger than the average deficit in the main results for total energy use (− 17%). Interestingly, a similar negative relationship exists between the size of the deficit and energy efficiency (+ 19% for AB, − 68% for FG). This result suggests that estimates of the EPG that do not account for electricity use in the home may be overstating the true EPG as they do not account for fuel switching. This is especially the case for homes with the lowest energy efficiency. The sample average deficit of 28% is in line with estimates of rebound effects observed in other contexts (Sorrell et al. 2009).

**Table 21:** Difference between annual actual (AQ) and theoretical (TQ) gas use (AA_Relative_)

	*n*	Mean AQ	Mean TQ	Difference	% Difference	SE	*P* value
AQ_All_ – TQ	19,251	9452.773	13,148.1	− 3695.331	− 28%	64.591	0***
*EPC grade*							
AB	2601	8979.353	7570.791	1408.562	19%	134.252	0***
C	8269	9279.777	10,825.76	− 1545.985	− 14%	77.938	0***
D	4835	9628.3	14,353.03	− 4724.732	− 33%	114.68	0***
E	2051	9990.076	18,132.59	− 8142.514	− 45%	189.71	0***
FG	1495	9928.487	24,961.51	− 15,000	− 60%	303.971	0***
*Dwelling type*							
Apartment	1674	6945.365	11,595.13	− 4649.767	− 40%	177.632	0***
Detached	2316	12,413.54	19,384.96	− 6971.412	− 36%	259.594	0***
Semi-detached	6905	9990.711	14,008.02	− 4017.31	− 29%	105.639	0***
Terrace	8356	8689.943	11,019.98	− 2330.038	− 21%	86.446	0***

^***^P < 0.01, **P < 0.05, *P < 0.10. units in kWh/year. Sample features 9923 observations of 1 year of actual GAS use and a further 9328 observations from the same sample of houses with a second year of actual GAS use. A test of equality of medians (using signtest STATA command (Snedecor & Cochran, 1989), confirms the same differences exist

Although Table 21 made a suitable comparison between gas energy use and the EPC, Table 22 is difficult to interpret because electricity use in the home was never intended to be the sole determinant of the EPC (which reflects space and water heating). As such, the average deficits are large negative values. This makes sense, as electricity is more commonly used as a secondary fuel in this sample, in association with gas heating.

**Table 22:** Difference between annual actual (AQ) and theoretical (TQ) electricity use (AA_Absolute_)

	*n*	Mean AQ	Mean EPC	Difference	% Difference	SE	P value
AQ_All_ – TQ	19,251	4134	13,148	− 9014	− 69%	53	0***
EPC grade							
AB	2601	4232	7571	− 3339	− 44%	81	0***
C	8269	4321	10,826	− 6505	− 60%	51	0***
D	4835	4018	14,353	− 10,300	− 72%	81	0***
E	2051	3793	18,133	− 14,300	− 79%	147	0***
FG	1495	3777	24,962	− 21,200	− 85%	272	0***
Dwelling type							
Apartment	1674	3198	11,595	− 8397	− 72%	126	0***
Detached	2316	4726	19,385	− 14,700	− 76%	222	0***
Semi-detached	6905	4257	14,008	− 9751	− 70%	82	0***
Terrace	8356	4056	11,020	− 6964	− 63%	67	0***

^***^P < 0.01, **P < 0.05, *P < 0.10. Sample of 9923 homes with 9923 observations of 1 year of actual electricity use and a further 9328 observations for the same houses with a second year of observed actual electricity use. A test of equality of medians (using signtest STATA command (Snedecor & Cochran, 1989), confirms the same differences exist

## Data Availability

I wish to highlight that the household-level consumption data used for this paper was provided under a non-disclosure agreement with an energy utility and unfortunately cannot provided for submission in a repository or as an electronic resource. The dataset of household-level dwelling characteristics and expected energy efficiency is publicly available from the Sustainable Energy Association of Ireland (SEAI) online (https://ndber.seai.ie/BERResearchTool/Register/Register.aspx).

## Footnotes

1 See https://ec.europa.eu/energy/en/content/introduction-11

2 Bunching is defined by Hyland et al. (2016) as ‘an excess frequency of homes on the favourable side of a threshold accompanied by a much-reduced frequency on the unfavourable side of that threshold.’.

3 The Energy Performance Gap can be considered within the broader theory of the Energy Efficiency Gap (Jaffe and Stavins 1994) which considers the under-adoption of energy-efficient goods with a positive net present value at the societal level.

4 Studies of domestic energy use are complicated by such ‘rebound’ effects, where improvements in energy efficiency lower the cost of energy services, thus increasing energy use (Sorrell et al., 2009).

5 The Sustainable Energy Authority Ireland (SEAI) promotes energy efficiency in Ireland and manages the Irish EPC certification process.

6 See https://www.rte.ie/eile/brainstorm/2017/1124/922516-missing-climate-and-energy-targets-will-cost-ireland-millions/

7 See https://www.seai.ie/data-and-insights/seai-statistics/key-statistics/residential/#:~:text=For%202018%20we%20estimate%20that,%2C%20and%202%25%20for%20cooking.

8 The term ‘bimonthly’ denotes a period of 2 months. This is not to be confused with the ‘twice-monthly’ frequency.

9 Appendix 6 performs a robustness check of annual results, split by subsample and finds no major differences.

10 Primary energy use includes energy delivered to the home and an overhead for energy lost in generation and transmission.

11 Appendix 1 provides more detail on the construction of the dependent variable.

12 This is detailed further in Appendix 2.

13 Available on the European Climate Assessment & Dataset http://eca.knmi.nl/dailydata/customquery.php

14 Appendix 5 features a robustness check that splits data for each full year of actual energy use (Y1, Y2).

15 The Irish regulator reference values for average energy use are discussed in Appendix 4.

16 Appendix 7 confirms the relationship persists using a categorical version of theoretical energy use.

17 There were 26,154 electricity customer switches on average each month in 2017 (CRU, 2018).
